# Evidence of Insulin-Sensitizing and Mimetic Activity of the Sesquiterpene Quinone Avarone, a Protein Tyrosine Phosphatase 1B and Aldose Reductase Dual Targeting Agent from the Marine Sponge *Dysidea avara*

**DOI:** 10.3390/pharmaceutics15020528

**Published:** 2023-02-04

**Authors:** Marcello Casertano, Massimo Genovese, Alice Santi, Erica Pranzini, Francesco Balestri, Lucia Piazza, Antonella Del Corso, Sibel Avunduk, Concetta Imperatore, Marialuisa Menna, Paolo Paoli

**Affiliations:** 1 Department of Pharmacy, University of Naples “Federico II”, Via D. Montesano 49, 80131 Naples, Italy; 2 Department of Experimental and Clinical Biomedical Sciences, University of Florence, Viale Morgagni 50, 50134 Florence, Italy; 3 Biochemistry Unit, Department of Biology, University of Pisa, Via S. Zeno 51, 56123 Pisa, Italy; 4 Interdepartmental Research Center for Marine Pharmacology, Via Bonanno 6, 56126 Pisa, Italy; 5 Medical Laboratory Programme, Vocational School of Health Care, Mugla University, Marmaris 48187, Turkey

**Keywords:** marine natural products, *Dysidea avara*, sesquiterpene-type quinones, avarone, aldose reductase, protein tyrosine phosphatase 1B, dual target inhibitors, diabetes mellitus

## Abstract

Type 2 diabetes mellitus (T2DM) is a complex disease characterized by impaired glucose homeostasis and serious long-term complications. First-line therapeutic options for T2DM treatment are monodrug therapies, often replaced by multidrug therapies to ensure that non-responding patients maintain target glycemia levels. The use of multitarget drugs instead of mono- or multidrug therapies has been emerging as a main strategy to treat multifactorial diseases, including T2DM. Therefore, modern drug discovery in its early stages aims to identify potential modulators for multiple targets; for this purpose, exploration of the chemical space of natural products represents a powerful tool. Our study demonstrates that avarone, a sesquiterpene quinone obtained from the sponge *Dysidea avara*, is capable of inhibiting in vitro PTP1B, the main negative regulator of the insulin receptor, while it improves insulin sensitivity, and mitochondria activity in C2C12 cells. We observe that when avarone is administered alone, it acts as an insulin-mimetic agent. In addition, we show that avarone acts as a tight binding inhibitor of aldose reductase (AKR1B1), the enzyme involved in the development of diabetic complications. Overall, avarone could be proposed as a novel natural hit to be developed as a multitarget drug for diabetes and its pathological complications.

## 1. Introduction

Insulin, a peptide hormone secreted by β-cells of the pancreas, represents the principal regulator of glycemic homeostasis. Diabetes mellitus (DM) is characterized by hyperglycemia that results from decreased tissue response to insulin and/or inadequate insulin secretion in the complex pathways of hormone action. The traditional classification of diabetes comprises four categories: gestational diabetes, Type 1, Type 2, and specific types of diabetes caused by other factors. Long-term diabetic complications are related to chronic hyperglycemia and consist of cataracts, cardiovascular complications, nephropathy, retinopathy, and neuropathy [1]. According to the World Health Organization (WHO) data, today, more than 420 million people are living with diabetes worldwide. This number is estimated to rise to 570 million by 2030 and 700 million by 2045 [2] and, contrary to the other main non-communicable diseases, the premature mortality for diabetes has increased by 5% from 2000 to 2016 [3]. Furthermore, it is thought that all these predictions could be negatively altered by COVID-19 [4]. The WHO and the International Diabetes Federation are working together to warrant the best quality of life possible for people with diabetes worldwide. In this context, there is a steadily developing need to increase and improve DM treatment approaches by discovering new, more effective and safer therapeutic options. This is relevant today more than ever, as unfortunately, the poor outcome from COVID-19 of people affected by DM has been shown dramatically.

Developing new therapeutics for DM treatment/prevention strongly relies on a detailed knowledge of the molecular mechanisms and cellular pathways underlying the onset of insulin resistance. The insulin signal pathway determines the activation of the insulin receptor (IR)/insulin receptor substrate (IRS)/phosphatidylinositol 3-kinase (PI3K)/protein kinase B (Akt) promoting the uptake of peripheral glucose through GLUT transporters, glycogen synthesis, and other metabolic effects [1]. Among organs that are targets of insulin, adipose tissue responds to endocrine and metabolic signals by modulating a variety of adipokines and cytokines that can affect locally and systemically [5]. Dysfunction in this crosstalk may become deleterious, causing low-grade inflammation and insulin resistance [5]. Aldose Reductase (AKR1B1) and Protein Tyrosine Phosphatase 1B (PTP1B) represent two insulin-resistance-related targets involved in the development of type 2 diabetes mellitus (T2DM) and its chronic complications. AKR1B1 is a cytosolic enzyme that catalyzes the reduction of aldoses to the corresponding alcohols [6], thus being the rate-limiting step of the polyol pathway. This leads to several metabolic alterations related to an increase in NF-kB expression and ROS generation, resulting in oxidative stress, induction of an inflammatory response, and the impairment of the glutathione reductase-dependent recovery of reduced glutathione. Under these conditions, a ROS increase induces lipid peroxidation with the consequent formation of cytotoxic aldehydes, such as 4-hydroxy-trans-2-nonenal (HNE) and its glutathione conjugate. AKR1B1 catalyzes reduction of this last metabolite to the corresponding alcohol (GS-DHN) [7], which also promotes the expression of NF-κB. Thus, the enhanced activity of AKR1B1 promotes not only the onset of oxidative stress linked to insulin resistance but also the long-term complications linked to T2DM [6,8,9]. PTP1B is one of the most important enzymes involved in the regulation of the insulin signaling pathway as it catalyzes the dephosphorylation of phosphotyrosine residues of IR and IRS, thus damping the receptor response following insulin binding [10]. PTP1B overexpression has been related to the onset of insulin resistance, and knockout mice for PTP1B do not develop insulin resistance even if they are fed a high-fat diet [11]. Moreover, inside neurons of the arcuate nucleus of the hypothalamic region, PTP1B catalyzes dephosphorylation of the JAK2 tyrosine kinase associated with the leptin (an adipokine that promotes anorexigenic effects) receptor. Increased PTP1B activity in the hypothalamic region is responsible for the upregulation of orexigenic signals, thereby promoting hyperphagia that, in turn, causes obesity. Both PTP1B and AKR1B1 are, thus, currently emerging as suitable targets to be modulated with drug-like small molecules to discover new therapeutics for insulin resistance and obesity [12,13]. Moreover, in a poly-pharmacology strategy, dual inhibitors of PTP1B and AKR1B1 could be used as multitarget drugs to treat both conditions of insulin resistance and chronic complications associated with T2DM [14].

**Figure 1:**
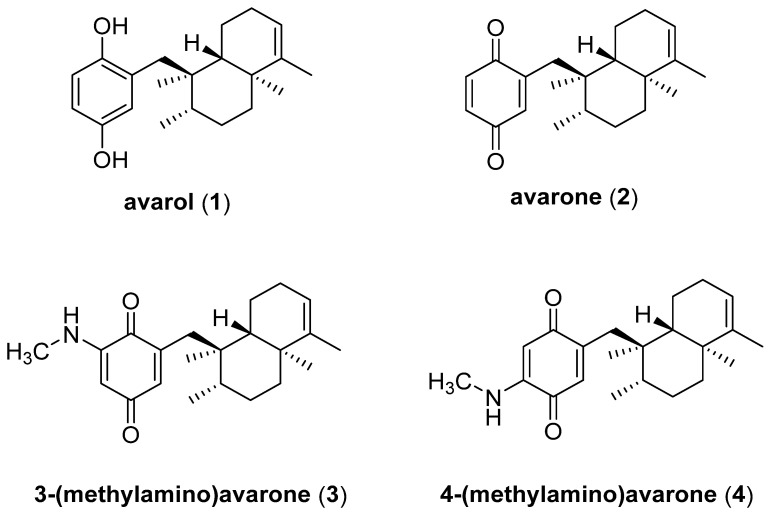
Structures of the marine-derived hydroquinone avarol (**1**), its oxidized form avarone (**2**), and the methylamine derivatives of avarone (**3** and **4**) isolated from the sponge *D. avara*.

Natural Products (NPs) are an excellent resource of chemical diversity for drug discovery [15,16]. In addition to providing bioactive chemical scaffolds for medicinal chemistry optimization, NPs are ideal chemical probes to determine the biochemical pathways underlying a given disease and to discover novel drug targets. Natural quinone/hydroquinone (Q/HQ) compounds are privileged scaffolds, due to their capability to participate in important biological redox processes and to affect radical production, resulting in a wide variety of pharmacological effects [17,18,19,20,21,22,23,24], as well as due to their high prevalence in the environment [17,18]. Recently, we undertook research to identify new chemotypes active as dual inhibitors of PTP1B and AKR1B1 using NPs of marine origin [25,26]. A significant inhibitory activity against several phosphatases, including PTP1B, by sesquiterpenes quinones of marine origin was reported [24,27]. On the other hand, antidiabetic properties due to aldose reductase inhibition were evidenced for benzoquinones, anthraquinones, and naphthoquinones, which were shown to all be noncompetitive inhibitors of the enzyme [17]. We had the opportunity to investigate a sample of the marine sponge *Dysidea avara* (Schmidt, 1862) collected along the coast of Turkey (İzmir Bay, Aegean Sea) and we isolated the sesquiterpene Qs/HQs avarol (**1**), its oxidized form avarone (**2**), and methylamine derivatives of avarone (**3** and **4**, Figure 1) [19]. The great potential for new drug discovery of these compounds as well as other structurally related secondary metabolites (e.g., ilimaquinone [28,29]) is evidenced by the many biological effects reported, including anti-tumor, anti-inflammatory, anti-mutagenic, anti-bacterial, anti-viral, anti-psoriatic, anti-parasitic, radical scavenger, inhibition of the lipid peroxidation, and anti-biofouling activities [19,20,21,22,30,31,32,33,34,35,36,37]. In the present study, we evaluated the inhibition effects of the sesquiterpene quinones **1**–**4** against PTP1B and AKR1B1 enzymes. The obtained results are encouraging for the possibility of optimizing and developing the sesquiterpene quinone scaffold of avarone (**2**) for the discovery of newly designed multiple ligands (DMLs) for the treatment of T2DM.

## 2. Materials and Methods

### 2.1. Materials

The commercial solvents and deuterated solvents used for extraction and HPLC purification were purchased from Sigma-Aldrich (Saint Louis, MO, USA) and were used without further purification. TLC (Silica Gel 60 F254, plates 5 × 20, 0.25 mm) were purchased from Merck (Kenilworth, NJ, USA). NADPH and L-idose were from Carbosynth (Compton, UK). *P*-nitrophenylphosphate, pNPP and p-IR β subunit Y1162/1163 (sc-25103-R), β-actin clone C-4 (sc-47778), and anti-rabbit and anti-mouse secondary antibodies were from Santa Cruz Biotechnology (Dallas, TX, USA); IRβ subunit antibody clone CT-3 (MABS65) was from Merck-Millipore (Burlington, MA, USA); p-protein kinase B (pAkt) (9271S) and Akt (9272S) antibodies were from Cell Signaling Technology (Danvers, Massachusetts, USA). All other reagents, unless otherwise specified, were obtained from Merck Life Science S.r.l. (St. Louis, MO, USA). High-resolution electrospray ionization−mass spectrometry (ESI-MS) was performed on a Thermo LTQ Orbitrap XL spectrometer (Thermo-Fisher, San Josè, CA, USA). The MS spectra of compounds **1**–**4** were recorded by infusion into the ESI source using methanol (MeOH) as a solvent. The ^1^H (700 MHz) and ^13^C (175 MHz) NMR experiments were carried out on a Bruker Avance Neo spectrometer (Bruker BioSpin Corporation, Billerica, MA, USA); chemical shifts were reported in parts per million (ppm) and referenced to the residual solvent signal (CDCl_3_: *δ*_H_ = 7.26; *δ*_C_ = 77.0) [38,39]. High-performance liquid chromatography (HPLC) separation was achieved on Knauer K-501 and Shimadzu LC-10AT (Shimadzu, Milan, Italy) apparatuses both equipped with a Knauer K-2301 RI detector (LabService Analytica s.r.l., Anzola dell’Emilia, Italy).

### 2.2. Isolation of Sesquiterpenes ***1**–**4*** from the Sponge D. avara

The hydroquinone avarol (**1**) and its oxidized form, the quinone avarone (**2**), were obtained in pure form by extraction of a sample of the Aegean sponge *D. avara* available at the Department of Pharmacy of the University of Naples Federico II. Indeed, a sample of the sponge (collected in Narlidere, Bay of Izmir, Turkey, 38°24′45 N 27°8′18 E) was taken from the existing collection of natural sources, and it was extracted according to our previously reported procedure [19,25,40,41]. Indeed, specimens of *D. avara* were thawed, homogenized, and first extracted with methanol (3 × 500 mL) at room temperature and, subsequently, with chloroform (3 × 500 mL), providing different extracts that were combined and concentrated in vacuo. The obtained suspension was dissolved in water, partitioned first with ethyl acetate and then with n-BuOH. The latter extract (800 mg after evaporation of the solvent as dark brown oil) was chromatographed on a RP-18 silica gel flash column, as reported in [19]. The most interesting fraction, labeled as A (23.2 mg), was eluted with MeOH/H_2_O 8:2 (v/v), showing the presence of diagnostic NMR signals related to hydroquinone/quinone derivatives. Accordingly, fraction A was subjected to HPLC purification on an RP-18 column (Luna, 5 μm C-18, 250 × 4.6 mm, flow rate = 1.00 mL/min), using MeOH/H_2_O 95:5 (*v/v*) as the mobile phase. This analysis afforded avarol (**1**, 6.2 mg, t_R_ = 5.1 min), and avarone (**2**, 7.7 mg, t_R_ = 9.0 min) in pure form. As for the ethyl acetate soluble material (550 mg), it was chromatographed by the open column on silica gel with the elution starting from 100% n-hexane and gradually increasing to 100% EtOAc. Then, it was followed by a step gradient of CH_2_Cl_2_:MeOH in different concentrations, and increased up to 100% MeOH. The fraction eluted with CH_2_Cl_2_:MeOH 8:2 (*v/v*), labeled as fraction B (13.6 mg), was chromatographed on an RP-18 column (Luna, 5 μm C-18, 250 × 4.6 mm, flow rate = 1.00 mL/min), with a mixture MeOH/H_2_O 9:1 (*v*/*v*), affording compounds **3** (2.2 mg, t_R_ = 14.4 min) and **4** (2.8 mg, t_R_ = 16.6 min) in the pure state. The identity of the isolated compounds **1***–***4** was confirmed by comparison of their spectroscopic properties (^1^H and HRESI-MS) with those reported in the literature [19,36,37,42]. Moreover, their purity (≥99.7%) was confirmed by HPLC analyses.

Avarol (**1**): yellow powder; ^1^H NMR (CDCl_3_) and HRESI-MS spectra are reported in Supplementary Materials (Figures S1 and S2); HRESI-MS *m/z* 315.2320 [M+H]^+^ (calcd. for C_21_H_31_O_2_: 315.2319).

Avarone (**2**): yellow powder; ^1^H NMR (CDCl_3_) and HRESI-MS spectra are reported in Supplementary Materials (Figures S3 and S4); HRESI-MS *m/z* 313.2166 [M+H]^+^ (calcd. for C_21_H_29_O_2_: 313.2162).

3-(methylamino)avarone (**3**): red powder; ^1^H NMR (CDCl_3_) and HRESI-MS spectra are reported in Supplementary Materials (Figures S5 and S6); HRESI-MS *m/z* 342.2428 [M+H]^+^, and *m/z* 364.2247 [M+Na]^+^ (calcd. for C_22_H_32_NO_2_: 342.2428).

4-(methylamino)avarone (**4**): red powder; ^1^H NMR (CDCl_3_) and HRESI-MS spectra are reported in Supplementary Materials (Figures S7 and S8); HRESI-MS *m/z* 342.2426 [M+H]^+^, and *m/z* 364.2245 [M+Na]^+^ (calcd. for C_22_H_32_NO_2_: 342.2428).

### 2.3. Enzymes Expression and Purification

The purification of human recombinant AKR1B1 was performed as previously described [43]. The specific activity of the purified enzyme was 5.3 U/mg. The purified enzyme, stored at −80 °C in 10 mM sodium phosphate buffer pH 7.0 containing 2 mM DTT and 30% (*w/v*) glycerol, was extensively dialyzed against 10 mM sodium phosphate buffer pH 7.0 before use. Human PTP1B coding sequence (1-302 aa) was cloned into the bacterial expression vector downstream and in frame with the polyHis tag. After expression, recombinant protein was purified using a chromatographic column loaded with Ni-NTA Resin (Thermo Fischer Thermo Fisher Scientific 168 Third Avenue Waltham, MA, USA). The fractions containing PTP1B were collected, concentrated, and injected into a Superdex G75 column equilibrated with Tris-HCl buffer pH 8.0 containing 150 mM NaCl and 1 mM mercaptoethanol. The fractions containing PTP1B were collected and concentrated; the purity of preparation was evaluated using SDS-PAGE. The PTP1B preparation was split into fractions of 0.5 mL volume; each fraction was stored at −80 °C.

### 2.4. Determination of Enzymatic Activity

The AKR1B1 activity was determined at 37 °C, as previously described [44], by following the decrease in absorbance at 340 nm linked to NADPH oxidation (ε340 = 6.22 mM*^−^*^1^.cm*^−^*^1^) through a Biochrom Libra S60 spectrophotometer. The standard assay mixture (final volume 0.7 mL) was contained in 0.25 M sodium phosphate buffer pH 6.8, 0.18 mM NADPH, 0.4 M ammonium sulfate, 0.5 mM EDTA, and 4.7 mM GAL. One unit of enzyme activity is the amount of enzyme that catalyzes the conversion of 1 µmol of substrate/min in the above assay conditions. The same assay conditions were adopted in the inhibition studies using L-idose as the substrate at the indicated concentrations. PTP1B activity was determined using p-nitrophenylphosphate (pNPP) as the synthetic substrate. The pNPP was dissolved in 0.075 M ββ-dimethylglutarate buffer, pH 7.0, containing 1 mM dithiothreitol. Each assay was carried out at 37 °C in 1 mL of assay buffer. The reactions were started by diluting an aliquot of PTP1B preparation in the assay buffer; after 30 min, the reactions were stopped by adding 2 mL of 0.2 M KOH solution. The amount of p-nitrophenol released was determined spectrophotometrically, reading the absorbance of solutions at 400 nm (ε400nm = 18 mM*^−^*^1^cm*^−^*^1^).

### 2.5. Determination of IC_50_ Values

Inhibitors were dissolved in DMSO; the solvent concentration in both the assay solution for AKR1B1 and PTP1B was kept constant at 0.5% (*v/v*) to avoid negative effects on enzymatic activity [45]. For IC_50_ evaluation, 10 mU of AKR1B1 in the presence of 1.6 mM L-idose was used. For each compound, at least five different concentrations (each at least in triplicate) were used. IC_50_ values were determined by non-linear regression analysis of experimental data using GraphPad Prism version 7.04 (GraphPad Software, San Diego, CA, USA). The IC_50_ values with respect to PTP1B were determined by measuring the enzymatic activity in the presence of an increasing concentration of compounds and a fixed substrate concentration (2.5 mM pNPP) corresponding to the K_M_ of PTP1B. For each compound, 12–15 different concentrations of each inhibitor were used. Each test was carried out in triplicate. Data obtained were normalized respect to the control sample. Experimental points obtained were fitted using the following equation:

$$\frac{V_{i}}{V_{0}}=\frac{\mathrm{Max}-\mathrm{Min}}{1+{\left(\frac{x}{{\mathrm{IC}}_{50}}\right)}^{\mathrm{slope}}}+\mathrm{Min}$$

where V_i_/V_0_ represents the ratio between the enzymatic activity calculated in the presence of the inhibitor (Vi) and the activity of the control sample (V_0_); “Max” and “Min” indicated the maximum and minimum of activity, respectively; IC_50_ represents the concentration of the inhibitor able to reduce the enzyme activity up to 50% with respect to the control, while the term “slope” represents the slope of the curve in the transition zone. The IC_50_ value was calculated by fitting experimental data with OriginPro 2021 software (OriginLab Corporation, One Roundhouse Plaza, Suite 303 Northampton, MA 01060, USA).

### 2.6. Kinetic Analyses

The kinetic characterization of avarone was performed considering the molecule as a “tight binding inhibitor”. AKR1B1 activity measurements obtained at each substrate concentration were fitted by nonlinear regression analysis to the Morrison equation reported below (Equation (1)) [46].

(1) $$v_{i}=1-\frac{\left(\left[E_{T}\right]+\left[I\right]+K_{i}^{\mathrm{app}}\right)-\sqrt{{\left(\left[E_{T}\right]+\left[I\right]+K_{i}^{\mathrm{app}}\right)}^{2}-4\left[E_{T}\right]\left[I\right]}}{2\left[E_{T}\right]}$$

This allowed the evaluation of $K_{i}^{\mathrm{app}}$. In order to obtain the inhibition constants K_i_ (dissociation constant of the EI complex) and $K_{i}^{\prime }$, (dissociation constant of the EIS complex), the $K_{i}^{\mathrm{app}}$ values were plotted against substrate concentration and fitted by nonlinear regression analysis to the following equation:

(2) $$K_{i}^{\mathrm{app}}=\left(\left[S\right]+{\;K}_{M}\right)/\left(K_{M}/K_{i}+\left[S\right]/K_{i}^{\prime }\right)$$

Equation (2) refers to a general case of a tight binding non-competitive inhibition model. The K_M_ value for L-idose was 3.9 mM [43]. The ability of different compounds to act as reversible AKR1B1 inhibitors was evaluated as described in [47]. The ability of avarone to act as a reversible or as a non-reversible inhibitor was evaluated by dilution assay [47]. The mechanism of action of avarone was evaluated by analyzing the effect of increasing avarone concentrations on the main kinetic parameters (K_M_ and V_max_) of PTP1B. Data obtained were analyzed using the Lineweaver–Burk plot. The value of K_i_ was determined using the appropriate equations.

### 2.7. Molecular Docking

Docking simulations were carried out with AutoDock Tools and AutoDock Vina (version 1.5.6rc3) [48] using the 2HNP structure of PTP1B and 7f5n of TC-PTP obtained from Protein Data Bank. Missing residues were identified and restored using MODELLER 10.2 [49]. Ions, co-crystallized ligan, water molecules, and co-factors were removed from the structure. Polar hydrogen and charges were added to the protein and missing atoms were restored; then, it was saved in PDBQT format. AutoGrid software was used for calculating the grid map of the interaction energies for various atoms of the ligand with the macromolecule. The grid box center and dimension were set at center_x: 46.969, center_y: 15.762, center_z: 1.839 and size_x: 64, size_y: 60, size_z: 56 for PTP1B. For TC-PTP, such data were set at center_x = −51.862, center_y = −51.862, center_z = 31.56 and size_x = 56, size_y = 60, size_z = 58. The dockings were performed using AutoDock Vina with an exhaustiveness of 30. The ligand structure was prepared and energy minimization was performed using DS viewer pro 6.0 (Accelrys, San Diego, CA, USA); the structure was saved in PDB format. Graphical representations of docked structures were performed using UCSF Chimera [50]. Two-dimensional representations of interactions were performed using Ligplot plus (version 2.2.5, Velizy-Villacoublay, Cambridge, United Kingdom) [51] and using Biovia Discovery Studio Visualizer [52].

### 2.8. Cell Cultures

The C2C12 cell line was grown in DMEM medium (high glucose, 4500 mg/L) supplemented with 10% FBS (Fetal Bovine Serum (FBS, Euroclone, Milan, Italy)), 2 mM glutamine, 100 U/mL of penicillin, and 100 μg/mL of streptomycin, under controlled temperature (37 °C), humidity, and CO_2_ concentration (5% CO_2_) conditions. When cells reached 70–80% confluence, cells were detached and part of these transferred to a new plate to favor their propagation.

### 2.9. Ex Vivo Assays

To evaluate the impact of avarone (2) on the insulin signaling pathway, C2C12 cells were plated on P35 dishes and grown until 70% confluence. Then, cells were washed with phosphate-buffered saline (PBS) and incubated for 24 h in the presence of a starvation medium (DMEM high glucose containing 2 mM glutamine, 100 U/mL of penicillin, and 100 μg/mL of streptomycin). After this time, cells were stimulated for 30 min with 10 nM insulin (Humulin R, Ely Lilly, Italy) with avarone (2) or the insulin-avarone combination. After this time, cells were washed with cold PBS and lysed using 100 µL of 1X Laemmli sample buffer solution (50 mM Tris-HCl pH 6.8, 2% SDS, 10% glycerol, 1% β-mercaptoethanol, 12.5 mM EDTA, 0.02% bromophenol blue). Cell proteins were withdrawn, transferred in an Eppendorf tube and boiled for 5 min. All samples were stored at −20 °C before the analysis. Analysis of samples was carried out using SDS-PAGE; after electrophoresis, proteins were transferred on a PVDF membrane by Western blot. Phosphorylation levels of the insulin receptor β chain and of the kinase Akt were evaluated using specific antibodies: IR β subunit, clone CT-3 (MABS65) was from Santa Cruz Biotechnology (Santa Cruz, CA, USA); pIR β subunit, Y1162/1163 (sc-25103-R) was from Merck-Millipore (Burlington, MA, USA). Akt (9272S) and p-Akt (9271S) antibodies were from Cell Signaling Technology (Danvers, MA, USA). Finally, β-actin and clone C-4 (sc-47778) were from Merck-Millipore (Burlington, MA, USA). Anti-rabbit and anti-mouse secondary antibodies were from Santa Cruz Biotechnology (Santa Cruz, CA, USA). Detection was performed using Clarity western ECL substrates (BioRad Laboratories, Inc. 1000 Alfred Nobel DriveHercules, CA, USA). Quantitation of the images obtained was carried out using iBright Analysis Software (Thermo Fisher Scientific 168 Third Avenue Waltham, MA, USA, 02451). Glucose uptake was evaluated as previously described [26]. Briefly, 70% confluent C2C12 cells were starved and then stimulated for 30 min with 10 nM insulin, avarone, or the insulin-avarone combination. After this time, cells were washed with PBS and incubated for 3 h with starvation medium containing 40 μM 2-NBDG (Invitrogen). Then, cells were washed with PBS, trypsinized, pelleted by centrifugation (1000× *g* for 5 min), and resuspended in 500 μL of PBS before the analysis. Intrinsic and 2-NBDG-derived cell fluorescences were determined using a flow cytometer (FACSCanto II, BD Biosciences, San Jose, CA, USA). For each sample, 1 × 104 events were acquired. Quantitative analysis was carried out using FlowJo software (FlowJo^TM^ v10. BD FlowJo™ Software for Windows Version 10. Ashland, OR: Becton, Dickinson and Company; 2021).

### 2.10. Seahorse XFe96 Metabolic Assays

C2C12 cells (3 × 104 cells/well) were placed in XFe96 cell culture plates. After 24 h of incubation at 37 °C, cell growth medium was substituted with an XF base medium supplemented with 2 mM glutamine, 1 mM sodium pyruvate, and 10 mM glucose, pH 7.4. The plate was then transferred in a non-CO_2_ incubator for 1 h at 37 °C to pre-equilibrate the cells before analysis. An XF Mito Stress Test was performed to assay the cells’ ability to exploit mitochondrial oxidative metabolism, according to the manufacturer’s instructions [53]. The oxygen consumption rate (OCR) was evaluated after the injection of a sequence of compounds that interfere with the electron transport chain: oligomycin (1 μM), carbonyl cyanide-4 (trifluoromethoxy) phenylhydrazone (FCCP) (1 μM), and Rotenone/Antimycin A (0.5 μM). All data were normalized on the protein content of each well.

## 3. Results

### 3.1. Bioprospecting of the Sponge D. avara for the Isolation of Compounds ***1**–**4***

The redox couple avarol (**1**) and avarone (**2**) as well as its methylamino derivatives, compounds **3** and **4**, were obtained in pure form by extraction of a sample of the Aegean sponge *D. avara* from the existing biological samples collection available at the Department of Pharmacy of University of Naples Federico II. Compounds **1**–**4** were isolated and purified according to our previously described procedure [25,40], and unequivocally identified by comparison of their HRMS and NMR data with those reported in the literature (Figures S1–S8). Solvents (ethyl acetate, n-butanol, and water) with different polarity were used for the extraction of the available sample of the fresh thawed sponge, providing three different dry organic extracts. The n-butanol and ethyl acetate soluble material were separately subjected to chromatographic separation on an RP-18 and SiO_2_ open column, respectively, providing two different interesting fractions (A and B) as confirmed by a preliminary ^1^H NMR analysis. Indeed, the diagnostic protons’ signals related to hydroquinone and/or quinone-related compounds were recorded and, accordingly, HPLC purification followed by the comparison of our experimental spectroscopic data with those reported in literature were performed, affording compounds **1**–**4** (Figure 1) in pure form (see f Materials and Methods) [19,36,37,42].

### 3.2. Evaluation of Inhibitory Effects against PTP1B and AKR1B1 of Compounds ***1**–**4***, and Kinetic Analyses of Avarone (***2***)

**Figure 2:**
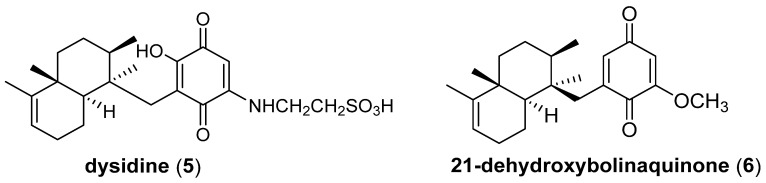
Structure of the marine sesquiterpene quinones dysidine (**5**) and 21-dehydroxybolinaquinone (**6**).

The four sesquiterpene-quinones (**1**–**4**, Figure 1) were analyzed to evaluate their ability to inhibit both PTP1B and AKR1B1. For each compound, the IC_50_ values were determined on both PTP1B and AKR1B1. The calculated values are reported in Table 1, together with the IC_50_ values of *p*-benzoquinone as the control [17,24], and dysidine (**5**, Figure 2), a natural sesquiterpene quinone PTP1B inhibitor [27]. The relative inhibition curves are reported in Figure S9. Vanadate and epalrestat were used as the reference inhibitor on PTP1B and AKR1B1, respectively.

As far AKR1B1 is concerned, compounds **1**–**4** result as reversible inhibitors. Avarone (**2**) is the most potent in the series and even more active than *p*-benzoquinone, the simplest natural quinone [17] (Table 1). As the IC_50_ value measured for avarone is of the same order of magnitude as the AKR1B1 concentration in the assay (78 nM, calculated on the basis of a molecular weight of 34 KDa), this compound is considered a “tight binding” inhibitor. Among the compounds analyzed, avarone (**2**) is also confirmed as the most powerful inhibitor of PTP1B, showing an IC_50_ value of 6.7 µM. Considering the high molar ratio (IC_50_/PTP1B concentration), avarone cannot be considered a tight binding inhibitor for PTP1B.

**Figure 3:**
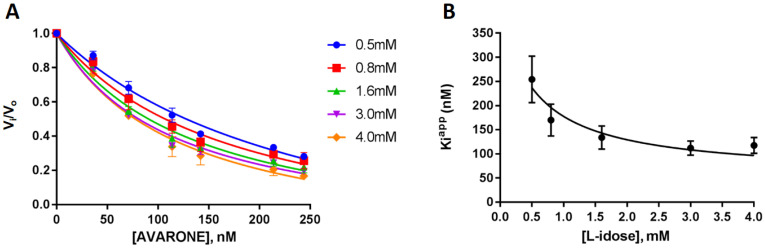
Kinetic characterization of avarone as AKR1B1 inhibitor. Panel A: the activity of the purified enzyme (10 mU) was measured at the indicated concentrations of avarone in the presence of the indicated L-idose concentrations. (**B**) Experimental data reported in (**A**) were fitted to the Morrison equation (see Materials and methods, Equation (1)). The obtained apparent inhibition constants, Ki^app^, were plotted against substrate concentration and fitted by nonlinear regression analysis to equation 2 (see Materials and methods) relative to a general case of tight binding non-competitive inhibition. Data were plotted using GraphPad 7.04 version.

The kinetic characterization carried out using recombinant AKR1B1 enzyme (see Methods for details) allowed the evaluation of K_i_ and K_i_’ values of 410 ± 94 and 55 ± 8 nM, respectively (Figure 3). Thus, avarone presents as a non-competitive mixed-type inhibitor of AKR1B1, with a preferential binding to the ES complex rather than the free enzyme.

**Figure 4:**
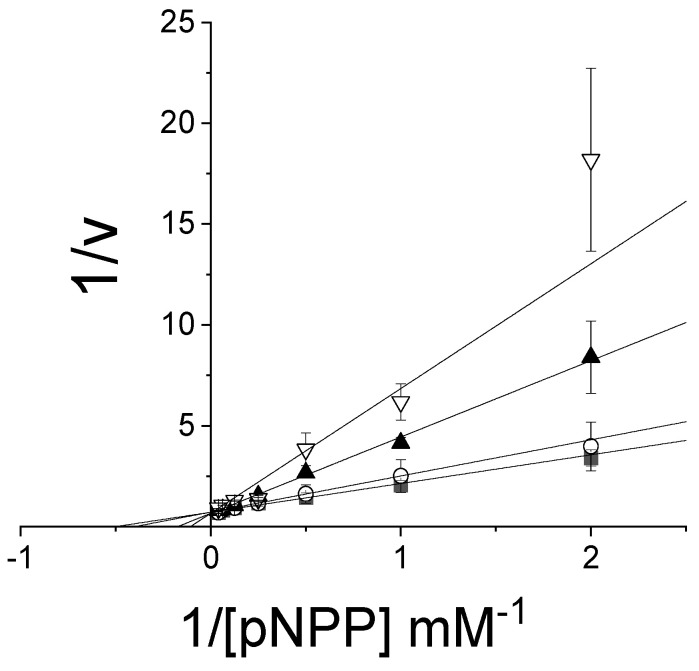
Impact of avarone on PTP1B-catalyzed hydrolysis of pNPP: the Lineweaver–Burk plot. Data reported in the figure were obtained by measuring the initial hydrolysis rate of pNPP at seven different concentrations, in the presence of increasing concentration of avarone. The concentrations of avarone used were: 0 µM, (●); 4 µM, (☐); 5.5 µM, (▲); 7 µM (▽). Each test was carried out in triplicate. Data reported in the figure represent the mean value ± SD.

Kinetic analyses were performed to define the mechanism of PTP1B inhibition by avarone, too. By dilution assay, we confirmed that avarone behaves as a reversible inhibitor of PTP1B (Figure S10). Moreover, to dissect the mechanism of inhibition of avarone, we analyzed the dependence of ^app^K_M_ and ^app^V_max_ from the concentration of the inhibitor. We observed that as the avarone concentration increases, the value of K_M_ increases, but the V_max_ substantially does not change (Figure S11); furthermore, the double reciprocal plot showed that the experimental points describe right lines intersecting one another in a point on the abscissa axis, suggesting that avarone behaves as a competitive inhibitor of PTP1B (Figure 4).

**Scheme 1:**
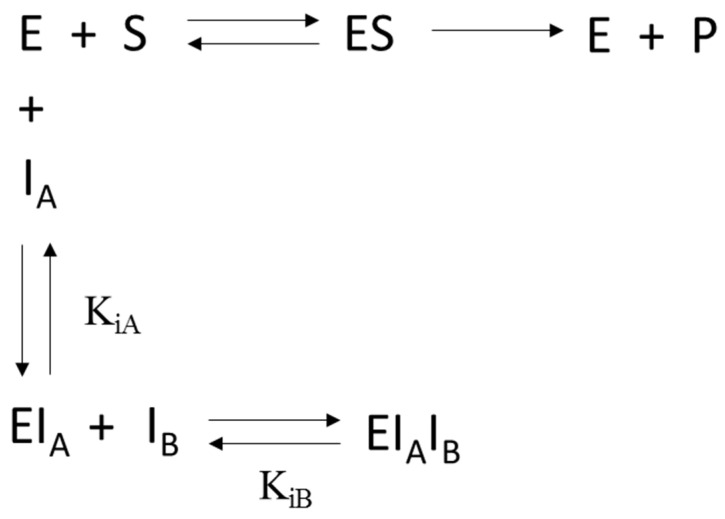
Mechanism of PTP1B inhibition by avarone. E: free enzyme; ES: Michaelis–Menten complex; P: product of reaction; I_A_ and I_B_: the two molecules of the inhibitor; EI_A_ and EI_A_I_B_: enzyme-inhibitor complexes; K_iA_ and K_iB_: enzyme inhibition constants for I_A_ and I_B_, respectively, i.e., the dissociation constants of the two corresponding enzyme-inhibitor/s complexes.

However, it is evident that K_M_ values increase non-linearly with increasing avarone concentration (Figure S11), raising the suspicion that the inhibition is determined by the binding of more than one avarone molecule within the enzyme site. Based on this evidence, we describe the mechanism of inhibition of avarone for PTP1B as a “cooperative pure competitive inhibition by two non-exclusive inhibitor molecules” (see Scheme 1).

**Figure 5:**
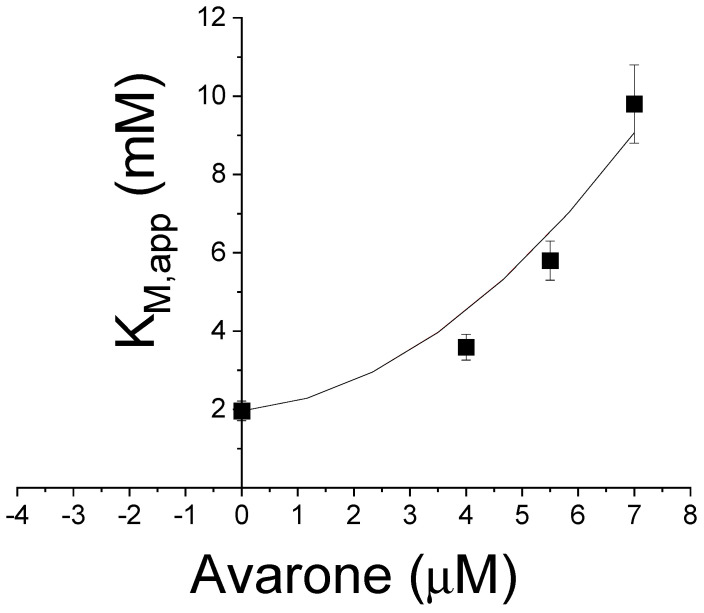
Determination of Ki_A_ and Ki_B_ for avarone. Dependence of K_M,app_ from the avarone concentration. Data obtained were fitted using Equation (3). Data fitting was obtained using OriginPlus 2021 software.

Such an inhibition model foresees that the first inhibitor molecule (I_A_) binds to the active site of the free enzyme and not to the ES complex. The second inhibitor molecule (I_B_) can only bind to EI_A_, generating the ternary complex (EI_A_I_B_). Considering the prevailing rapid equilibrium conditions, it was possible to derive Equation (3), which describes the dependence of K_M_ on the avarone concentration. Therefore, using ^app^K_M_ values determined at increasing avarone concentrations and Equation (1), we calculated both Ki_A_ (15.1 ± 1.4 µM) and Ki_B_ (1.0 ± 0.1 µM) values (Figure 5; details of the kinetic model are reported in Appendix A).

(3) $$K_{M,\mathrm{app}}=K_{M}\times \left\{1+\frac{\left[I\right]}{{\mathrm{Ki}}_{A}}+\frac{{\left[I\right]}^{2}}{{\mathrm{Ki}}_{A}\times {\mathrm{Ki}}_{B}}\right\}$$

### 3.3. PTP1B Docking Experiments

**Figure 6:**
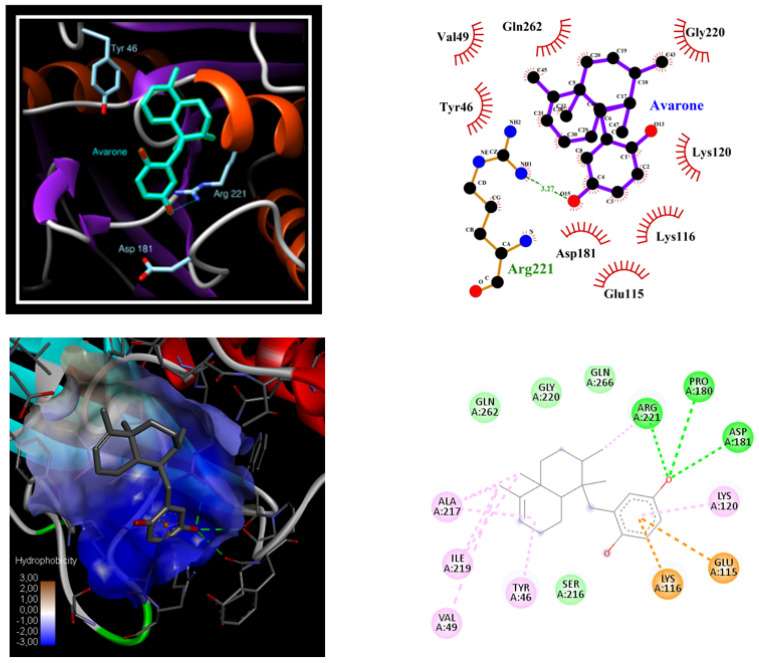
Docking of the avarone into the active site of PTP1B. The calculated free energy of binding was −7.8 kcal/mol. Images of avarone docked into active site of PTP1B were obtained using UCSF Chimera. Moreover, 2D representations of interactions were performed using Ligplot plus.

To better evaluate the interaction modality of avarone with PTP1B, in silico docking analyses were carried out with both compounds **1**–**5** and *p*-benzoquinone (Figure S12). In accordance with the kinetic analyses, we found that among all the compounds, avarone is the molecule that binds more tightly to the active site of the enzyme, showing a binding free energy of −7.8 kcal/mol (Table S1). The strength of the binding can be explained by the fact that the benzoquinone group of the avarone penetrates deeply into the active site of PTP1B and forms a hydrogen bond with one of the nitrogen atoms of the guanidine group of Arg 221. At the same time, the hexahydronaphthalene group is projected toward the outermost portion of the active site where it forms bonds with Tyr46, Val49, Glu115, Lys116, Lys120, Gly 220, and Gln262 (first pose), or with Trp179, Gly183, Ala217, Gly220, Gln262, and Gln266 (third pose) (Figure 6). None of other compounds can penetrate as deeply into the active site as avarone (Figure S12). Moreover, results of docking show that avarone can interact with the active site of the enzyme in multiple ways, also interacting with residues placed on the mouth of the active site (Figure S13). These results suggest that the active site of the enzyme can accommodate more than one molecule of avarone simultaneously, thus confirming the results obtained from kinetic tests, which show that the inhibitory activity of avarone is the result of a cooperative mechanism.

### 3.4. Specificity of PTP1B Inhibitory Activity of Avarone

To evaluate the capacity of avarone to specifically target the PTP1B enzyme, we determined the IC_50_ value for TC-PTP, another member of the tyrosine phosphatase family (Figure S14). The results of the analysis demonstrated that the affinity of avarone for TC-PTP is lower (5.8-fold) than that for PTP1B. This result is quite surprising if we consider the high similarity in the primary and tertiary structure of the two enzymes, and suggests that avarone interacts differently with the active sites of the two enzymes [55]. To confirm this hypothesis, the interaction mode of avarone with TC-PTP was analyzed by in silico docking analysis carried out using the crystallographic structure (7F5N) deposited in the PDB database [56]. The result of docking showed that avarone binds into the active site of TC-PTP assuming a different position with respect to that observed in the simulation carried out using the PTP1B enzyme (Figure S15).

### 3.5. Ex Vivo Assay

**Figure 7:**
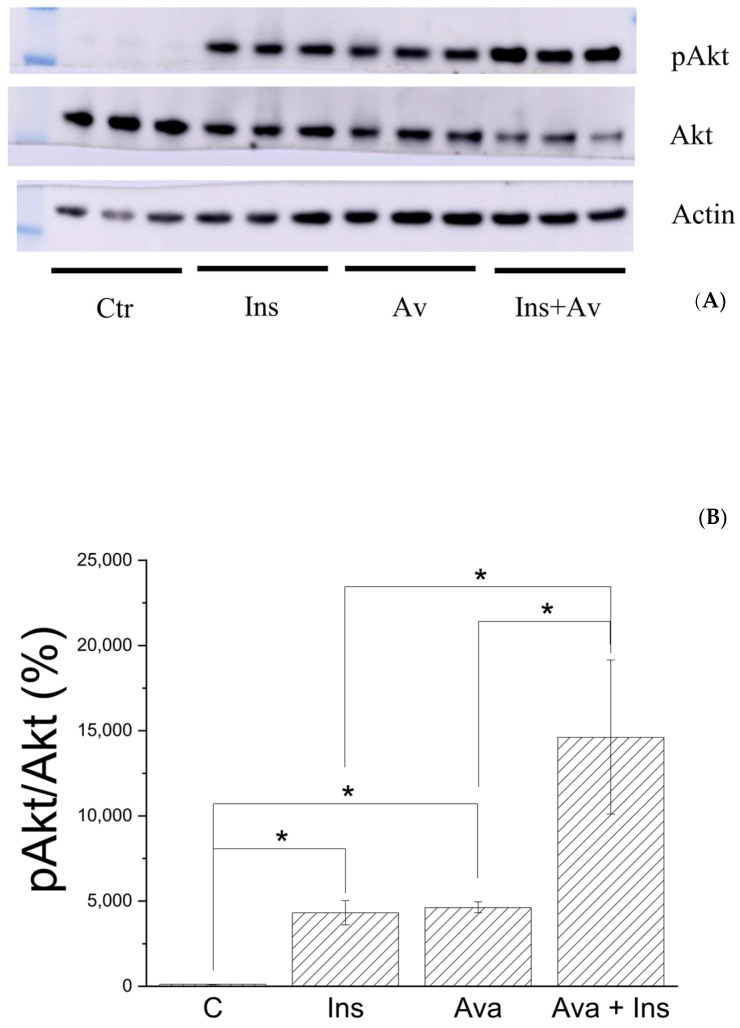
Effects of avarone on insulin signaling pathway. C2C12 cells were serum-starved and stimulated with 10 nM insulin (Ins), 25 µM of avarone (Ava), or with a combination of both (Ins + Ava) for 30 min at 37 °C. After, cells were washed, lysed, and analyzed to evaluate phosphorylation levels of Akt. Each test was carried out in triplicate. (**A**) Representative results of Western blot analysis; (**B**) quantitation of Western blot. Data were normalized with respect to control samples. Data reported in the figure represent the mean value ± SD. Student’s *t*-test was used to assess differences in experimental features. Statistical significance is annotated as follows: * *p* ≤ 0.05.

To evaluate the impact of avarone on the insulin signaling pathway, further tests were carried out using the myoblast cell line C2C12. Muscle cells were treated with insulin, avarone, or a combination of both. After 30 min, cells were lysed, and cells extracts were analyzed by Western blot to evaluate the phosphorylation levels of Akt (Figure 7 and Figure S16).

We observed that avarone causes an increase in Akt phosphorylation levels similar to that induced by insulin, while treatment with the insulin-avarone combination determines an enhanced effect, raising the levels of Akt phosphorylation to values higher than those observed after treatment with insulin alone. Such data suggest that avarone possesses both insulin-mimetic and insulin-sensitizing activity.

**Figure 8:**
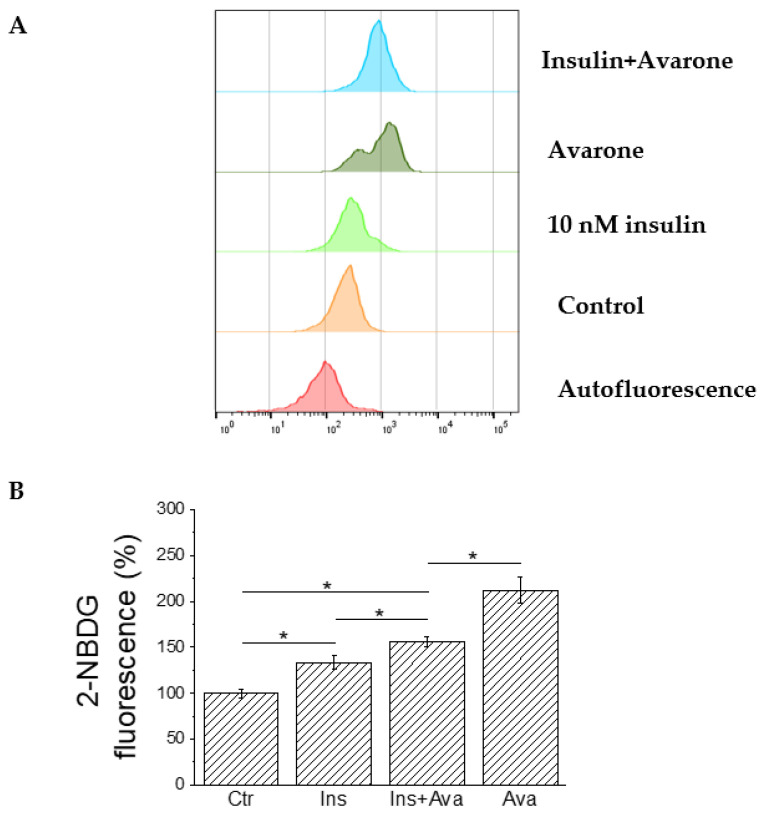
Glucose uptake assay. C2C12 cells were starved for 24 h and then treated with 10 nM insulin, 25 µM avarone, or a combination of both for 30 min at 37 °C. After, cells were washed and incubated for 3 h with 40 µM 2-NBDG. After, cells were washed with PBS, detached using trypsin, collected by centrifugation, and analyzed using a flow cytometer (FACSCanto II, BD Biosciences). (**A**) Flow cytometer analysis. For each experiment, 10,000 events were analyzed. Each test was carried out in quadruplicate. (**B**) Quantitation of fluorescence levels. The data shown in the figure represent the mean value ± SD. Student’s *t*-test was used to assess differences in experimental features. Statistical significance is annotated as follows: * *p* ≤ 0.05.

To confirm these results, we evaluated whether avarone can stimulate extracellular glucose uptake in C2C12 cells by incubating them with the fluorescent D-glucose analog 2-NBDG for 3 h and subsequently quantifying levels of intracellular fluorescence by flow cytometry analysis (Figure 8).

**Figure 9:**
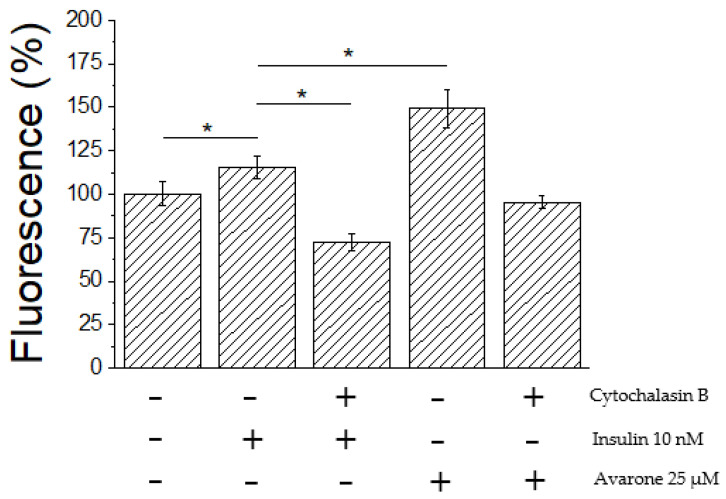
Impact of cytochalasin B on avarone-induced glucose uptake. C2C12 cells were starved for 24 h and then incubated for 2 h in the presence of 10 mM cytochalasin B. Then, cells were treated with 25 µM avarone or 10 nM insulin for 30 min at 37 °C. After, cells were washed and incubated for 3 h with 40 µM 2-NBDG and then analyzed using a flow cytometer (FACSCanto II, BD Biosciences). For each experiment, 10,000 events were analyzed. Each test was carried out in quadruplicate. The data shown in the figure represent the mean value ± SD. Student’s *t*-test was used to assess differences in experimental features. Statistical significance is annotated as follows: * *p* ≤ 0.05.

We observed that the levels of incorporated glucose significantly increase in cells treated with the insulin-avarone combination with respect to cells treated with insulin alone, confirming that avarone acts as an insulin-sensitizing agent. However, higher amounts of fluorescent glucose were also detected in cells treated with avarone alone (Figure 8), indicating that it is able to act also as an insulin-mimetic compound when administered alone. It is well known that insulin promotes glucose uptake, stimulating GLUT4 translocation to the plasma membrane of C2C12 cells [57]. Therefore, we speculated that avarone could favor the translocation/activation of GLUT4. To confirm this hypothesis, we evaluated the levels of fluorescent glucose incorporated in C2C12 cells pretreated or not with cytochalasin B, a potent inhibitor of GLUT4 activity (Figure 9) [58]. We observed that preincubation with Cytochalasin B strongly affects insulin-stimulated glucose uptake and abolishes the avarone-mediated glucose uptake. Taken together, these data confirm that avarone promotes GLUT4 translocation/activation.

### 3.6. Bioenergetics Analyses of C2C12 Cells Treated with Avarone

**Figure 10:**
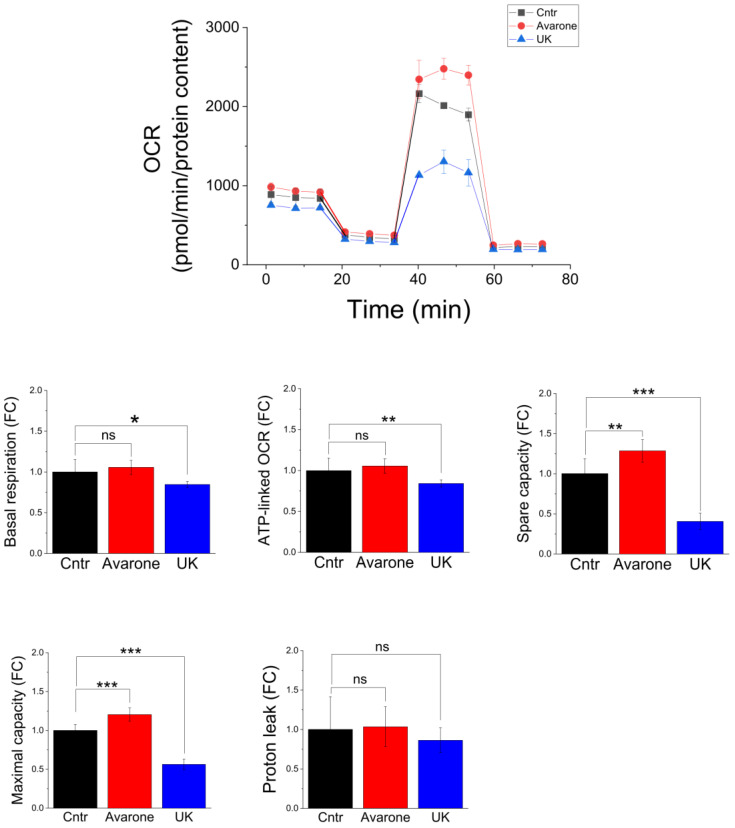
Results of Mito Stress Test assay. (**A**) C2C12 cells were subjected to Seahorse XFe96-Mito Stress Test analysis to evaluate oxygen consumption rate (OCR) in real time. OCR values were normalized on protein content. (**B**) Basal respiration, ATP-linked OCR, spare capacity, maximal respiration, and proton leak were calculated using data obtained from OCR plot after the administration of the adenosine triphosphate ATP synthase inhibitor oligomycin, the proton uncoupler carbonylcyanide *p*-trifluoromethoxyphenylhydrazone (FCCP), and the combination of the respiratory complex I inhibitor rotenone and the respiratory complex III inhibitor antimycin A. Each point reported in the OCR plot represents the medium value ± SEM obtained from independent replicates (n = 3 or 4). One-way ANOVA with Dunnett’s correction was used to assess differences in experimental features by using Prism 8 software (GraphPad). Statistical significance is annotated as follows: * *p* ≤ 0.01, ** *p* ≤ 0.001, *** *p* ≤ 0.0001, (*t*-test); ns, not significant; FC, fold change.

To evaluate whether avarone may affect mitochondrial metabolism, C2C12 cells were treated for 30 min with avarone and then analyzed for mitochondrial functionality by using the Seahorse XFe96 Analyzer (Figure 10). This approach allowed us to investigate the impact of this molecule on basal respiration, ATP production-related oxygen consumption, maximal respiration, spare respiratory capacity, non-mitochondrial respiration, and mitochondrial proton leak. Meanwhile, a comparative analysis was carried out treating C2C12 cells with UK5099, an inhibitor of the mitochondrial pyruvate carrier [59]. Interestingly, we found that treatment with avarone does not alter basal respiration, while UK5099 leads to a significant reduction in mitochondrial respiration, specifically affecting maximal respiration and ATP production-linked oxygen consumption (Figure 10A). This result is expected as UK5099 inhibits the mitochondrial pyruvate carrier, reducing mitochondrial pyruvate levels, NADH and FADH_2_ production, the flux of electrons through the mitochondrial electron transport chain, and consequently also the oxygen consumption. This finding also indicates that, unlike UK5099, the treatment with avarone does not impair the oxygen-dependent ATP production. Surprisingly, we found that treatment with avarone slightly increases both spare capacity and maximal respiration, without enhancing the proton leak rate (Figure 10B).

## 4. Discussion

A multitude of risk factors strongly influenced and determined T2DM as a complex disease that has reached alarming rates across the globe; some of these factors are modifiable by lifestyle changes (such as diet, physical activity levels, tobacco use), whereas others, such as age, ethnicity, and genetics, are nonmodifiable and adequate drug treatments are a key issue [60]. The failure of single-drug therapy against T2DM has encouraged the adoption of a multitarget treatment, mainly known as polypharmacology, switching from a one-target–one-drug model to a multiple-target approach. In this regard, searching for chemical scaffolds with a balanced activity toward different and selected targets is a great challenge, as identifying appropriate multifunctional molecules is quite difficult. Therefore, many researchers are concretely exploring the possibility of exploiting natural molecules, including those of marine origin, to discover novel DMLs for the treatment of T2DM. In the present study, we investigated the inhibitory effects against both PTP1B and AKR1B1 of four natural sesquiterpene Q/HQs (compounds **1**–**4**) isolated from the marine sponge *D. avara*.

Previously, the PTP1B inhibiting activity of related NPs has been reported; the two sesquiterpene quinones dysidine (**5**) and 21-dehydroxybolinaquinone (**6**) were shown to be PTP1B inhibitors with IC_50_ values of 6.7 μM and 39.5 μM, respectively (Figure 2) [27]. More in-depth studies revealed that the most active dysidine (**5**) was a reversible slow-binding PTP1B inhibitor, with moderate selectivity over other PTPs (TC-PTP and CD45). In addition, it has been shown that dysidine could activate the insulin signaling pathway, promote membrane translocation of the glucose transporter 4 (GLUT4) in CHO-K1 and 3T3-L1 cells, and increase glucose uptake in 3T3-L1 cells [24,27].

Thus, we tested the marine-derived compounds **1**–**4** against PTP1B; all compounds were found to be significantly active, although with different potency. The less active compound was the reduced form avarol (**1**), whereas avarone (**2**) resulted as the most active in the series, with an IC_50_ value of 6.7 μM. The methylamino group, either in 3 or 4 positions, present in compounds **3** and **4,** decreases its potency (Table 1). Moreover, avarone resulted as more active than both the simplest natural quinone, *p*-benzoquinone [24], and dysidine (**5**), a sesquiterpene hydroquinone acting as a competitive slow binding inhibitor of PTP1B [27]. The reason for the elevated inhibitory activity of avarone probably arises from its peculiar chemical structure, which allows it to assume several poses when it binds to the active site of PTP1B. It can penetrate deeply or interact with amino acids that form the active site crevice, suggesting that more than one molecule can bind the active site of PTP1B in the same moment. This hypothesis was also confirmed by results of kinetic analyses showing that two molecules bind to the active site of the enzyme with a different affinity. This led to the hypothesis that the first molecule, upon binding, causes a slight change in the structure of the enzyme’s active site that allows the second molecule to interact and bind more effectively to the enzyme’s active site. We can hypothesize that avarone is the only molecule capable of doing this among those analyzed.

Moreover, we observed that avarone shows greater specificity for PTP1B than TC-PTP. This is quite surprising considering that the two phosphatases share a high degree of similarity of the primary and tertiary structure of the active site [55]. The observed significant differences in the IC_50_ values of avarone against PTP1B (6.7 μM) over TC-PTP (39.2 μM), indicating a higher affinity for the former, could be attributed to slight differences in the conformation of the active site of the two enzymes that can influence the positioning of the inhibitor. Docking analyses carried out with both enzymes supported this hypothesis, indicating that the position of avarone can acquire different positions in the active site; we cannot exclude the possibility that the active site of PTP1B can host two molecules of avarone simultaneously. Furthermore, the free energy values calculated for the PTP1B-inhibitor complex are lower on average than those calculated for the TC-PTP inhibitor complex.

It is worth noting that avarone’s structure, as opposed to many competitive inhibitors identified and/or synthesized in recent years, lacks negative charged groups, such as phosphonates, sulphonates, or carboxylates. Such groups generally favor the interaction of inhibitors with the positive charged arginine, present at the bottom of the active site pocket of the PTPs. This may be considered a favorable feature of the structure of the new inhibitor; the presence of such negatively charged groups could indeed both act as an obstacle to the penetration of inhibitors into target cells and increase the probability that the inhibitors inhibit other members of the PTP family, generating severe side-effects. In fact, dysidine (**6**), featuring the ionizable sulfonic group in its structure, was shown to be a potent PTP1B inhibitor as avarone but with lower selectivity against PTP1B over TC-PTP [24]. Therefore, avarone represents an interesting prototype of a non-charged molecule that could be used as a model to design novel potent and bioavailable competitive inhibitors of PTP1B, thus helping to eliminate the concept that PTPs are undruggable enzymes [61].

Cellular assays revealed that avarone can activate the kinase Akt and stimulate glucose uptake in C2C12 cells either when administered alone or in combination with insulin. The kinase Akt acts downstream in the insulin receptor and is activated following insulin binding to its cognate receptor [62]. However, the degree of activation of the insulin receptor also depends on PTP1B activity, which dephosphorylates the insulin receptor, contributing to switch off the signal activated by insulin. Therefore, PTP1B is a key regulator of the insulin signaling pathway, and its overexpression, or unusual activation, contributes to the onset of insulin resistance [63]. Thus, by treating diabetic mice with PTP1B inhibitors, it is possible to boost insulin receptor activation, increase insulin sensitivity, and reduce blood glucose levels both in fasting conditions and after a meal [64]. The results of the tests on C2C12 cells (Figure 7) demonstrated that avarone, besides acting as an insulin sensitizer, also possesses insulin-mimetic activity, as suggested by its ability to induce the phosphorylation of Akt with the same intensity of insulin when used alone. Thus, we speculated that avarone could determine a strong activation of the Akt/PI3K pathway, triggering GLUT4 translocation to the plasma membrane also in the absence of insulin. Pre-treatment of C2C12 cells with cytochalasin B, a potent inhibitor of GLUT4 translocation, abolishes glucose uptake (Figure 9), confirming that avarone alone can boost migration of GLUT4 to the plasma membrane. However, it is interesting to note that avarone alone stimulates glucose uptake more efficiently than insulin itself; this evidence suggests that avarone acts through a complex mechanism that could also include the direct activation of GLUT4. In accordance with this hypothesis, previous studies reported that GLUT4 undergoes activation after its translocation to the plasma membrane of cells, and that both mechanisms contribute to the full stimulation of glucose uptake by insulin [65]. Therefore, to date, we cannot exclude that avarone may also act as a GLUT4 activator, enhancing, after exposure to the plasma membrane, the transporter’s ability to import glucose into muscle cells.

Bioenergetics analyses of C2C12 cells treated with avarone evidenced that the treatment increases the mitochondrial spare respiratory capacity (SRC) and the maximum respiration (MR). The increase in these parameters represents positive events because they witness the increased ability of cells to respond to stress stimuli, increasing the ATP production [66]. Based on literature data, we suppose that this effect can be another consequence of Akt activation as both SRC and MR are modulated by the PI3K/AKT/mTOR signaling pathway. According to this evidence, it has been reported that pharmacological inhibition of this pathway leads to a significant reduction in SRC; vice versa, the activation of the PI3K/AKT/mTOR signaling pathway, triggered by stimulation with different growth factors or through PTEN knockdown, results in an enhancement of glycolytic flux and of the SRC [67]. Therefore, we hypothesize that treatment of C2C12 cells with avarone could not only improve insulin sensitivity, and decrease blood glucose levels, but also positively impact mitochondrial activity. It is well known that the mitochondrial activity of muscle cells resulted as impaired in diabetic patients, leading to a reduction in fatty acid oxidation, promoting lipid accumulation in muscle cells, and favoring the development of the insulin resistance [68]. According to this finding, we hypothesize that avarone could contribute to revert the insulin resistance condition, improving mitochondrial activity in muscle cells of diabetic patients.

Based on the previously reported effects on aldose reductase of several quinone compounds [17], we tested compounds **1**–**4** against AKR1B1, the enzyme that is responsible for the conversion of glucose into sorbitol through the polyol pathway. Considering this action, AKR1B1 is considered a critical target to be inhibited to prevent the complications linked to an abnormal increase in the flux through the polyol pathway, as it occurs in hyperglycemic conditions [9]. Indeed, the overexpression of AKR1B1 in transgenic mice has been shown to accelerate the onset of diabetic cataracts [69] and exacerbate diabetic cardiomyopathy [70]. Blood glucose fluctuations and aldose reductase expression favor GSH depletion and apoptosis, thereby promoting the onset of retinopathy, and other diabetes-related complications [71]. All compounds **1**–**4** result in reversible inhibition of the AKR1B1 enzyme, and avarone (**2**) is the most potent in the series, again, with an IC_50_ value in the nanomolar range (0.078 μM). It shows a high affinity for AKR1B1 and can be considered a tight binding inhibitor of this enzyme. Interestingly, 3- or 4-substitution with the methylamino group in compounds **3** and **4** dramatically decreases the inhibition potency (73 and 62 μM). The kinetic characterization of **2** (Figure 3) allowed evaluation of Ki and Ki’ values (410 ± 94 and 55 ± 8 nM, respectively), highlighting avarone as a non-competitive mixed-type inhibitor of AKR1B1, with a preferential binding to the ES complex with respect to the free enzyme.

Definitively, we demonstrated that avarone is a potent inhibitor of both PTP1B and AKR1B1 enzymes, and, therefore, it can be considered as a multitarget agent.

**Figure 11:**
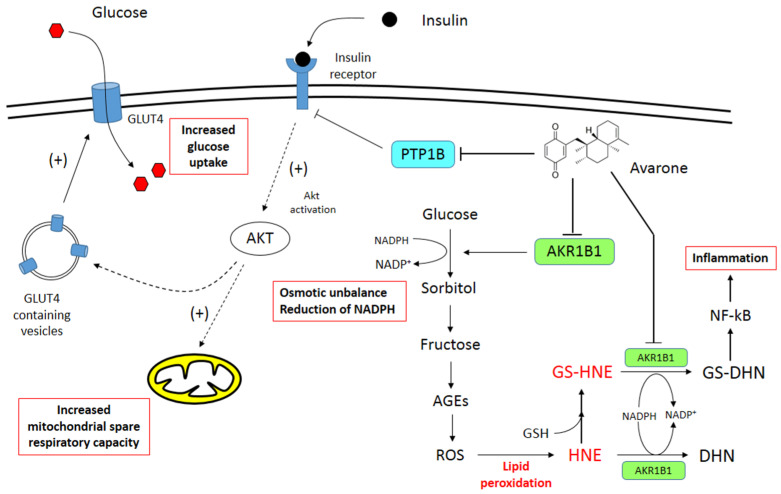
Graphical representation of avarone’s mechanism of action.

A schematic graphic representation of the putative molecular mechanism of avarone, which highlights its ability to interfere with the cellular events related to the activation of the insulin signaling pathway, is reported in Figure 11.

Furthermore, the potential of avarone as a lead compound is strongly enhanced by its very low cytotoxic effects; in fact, avarone exhibits an IC_50_ value of 62.19 μM when tested on human microvascular endothelial cells (HMEC-1) and results as not cytotoxic against human leukemia monocytic cells (THP-1) [19].

## 5. Conclusions

The thorough pharmacological characterization we conducted on avarone revealed that it is a potent and selective PTP1B inhibitor with both insulin-sensitizing and mimetic activity and that it displays positive effects on muscle cell mitochondria. These results reveal this natural hit as a promising candidate to be developed as an antidiabetic molecule. On the other hand, the intrinsic ability of avarone to act as a potent AKR1B1 inhibitor makes it an interesting scaffold molecule for developing a new series of potent and safe aldose reductase inhibitors. For these reasons, avarone could be proposed as a novel nontoxic natural hit to be developed for designing a multitarget drug for diabetes and its pathological complications. Our data confirm and reinforce the idea that the use of natural molecules, with their unique chemical structures and the inherent ability to act against multiple targets, play a crucial role in accelerating the discovery of new multitarget drugs.

## Figures and Tables

**Table 1:** Calculated IC_50_ values for PTP1B and AKR1B1.

Compound	IC_50_ (µM) ^a^	
	PTP1B	AKR1B1
avarol (**1**)	42.2 ± 18	0.52 ± 0.19
avarone (**2**)	6.7 ± 0.6	0.078 ± 0.017
3-methylaminoavarone (**3**)	15.2 ± 2.1	73 ± 15
4-methylaminoavarone (**4**)	21.6 ± 1.0	62 ± 8
*p*-benzoquinone	38.9 ± 5.2 ^¥^	1.93 ^‡^
dysidine (**5**)	6.7 ± 0.1 ^§^	-------
vanadate	0.4 ± 0.01 ^#^	-------
epalrestat	-------	0.13 ± 0.03

^a^ Data were expressed as IC_50_ ± SD. ^¥^ data from Zhang et al. [24]; ^‡^ data from Demir et al. [17]; ^§^ data from Li et al. [27]; ^#^ data from Maccari et al. [54].

## Data Availability

The tested compounds are available at the laboratory of the corresponding author.

## Supplementary Materials

The following supporting information can be downloaded at: https://www.mdpi.com/article/10.3390/pharmaceutics15020528/s1, Figure S1: HRESI-MS spectrum of avarol (**1**); Figure S2: ^1^H NMR spectrum of avarol (**1**) in CDCl_3_ (600 MHz); Figure S3: HRESI-MS spectrum of avarone (**2**); Figure S4: ^1^H NMR spectrum of avarone (**2**) in CDCl_3_ (600 MHz); Figure S5: HRESI-MS spectrum of 3-methylaminoavarone (**3**); Figure S6: ^1^H NMR spectrum of 3-methylaminoavarone (**3**) in CDCl_3_ (600 MHz); Figure S7: HRESI-MS spectrum of 4-methylaminoavarone (**4**); Figure S8: ^1^H NMR spectrum of 4-methylaminoavarone (**4**) in CDCl_3_ (600 MHz); Figure S9: Determination of the IC_50_ values for PTP1B and for AKR1B1; Figure S10: Dilution assay; Figure S11: Dependence of K_M_ and V_max_ from avarone (**2**) concentration; Figure S12: Docking of compounds **1**–**5** and of *p*-benzoquinone; Figure S13: Binding sites of avarone (**2**) identified by docking analyses; Figure S14**.** Determination of the IC_50_ values of avarone for PTP1B (A) and TC-PTP (B); Figure S15**.** Binding sites of avarone (**2**) identified by docking analyses with TC-PTP; Figure S16: Full Western blot of avarone on insulin signaling pathway; Table S1: Binding free energies of compounds **1**–**5** and of *p*-benzoquinone to PTP1B and TC-PTP.

## Appendix A

Steady-state kinetic analysis of the inhibition mechanism of avarone.

Based on our experimental data reported in the Section 3, we can hypothesize that PTP1B is able to bind two avarone molecules in the active site simultaneously. Furthermore, the hyperbolic dependence of KM on the avarone concentration suggests that the binding of the first molecule causes a distortion of the active site of the enzyme, thus facilitating the binding of the second avarone molecule. This model predicts that the two molecules bind with different affinity to the active site of the enzyme, with KiA > KiB, indicating that avarone shows a higher affinity for the EIA complex than the free enzyme (E).

The catalysed p-nitrophenylphosphate (pNPP) hydrolysis can be described as follow:

where E•pNPP is the Michaelis-Menten complex, and E-P is the covalent phosphoenzyme intermediate. By applying the steady state assumption to [E-P], we can assume:

(A1) $$k_{2}\cdot \left[E\cdot pNPP\right]=k_{3}\cdot \left[E-P\right]\;\left[E\cdot pNPP\right]=\frac{k_{3}}{k_{2}}\;\left[E-P\right]$$

By the max conservation lay

(A2) $${\left[E\right]}_{T}=\left[E\right]+\left[E\cdot pNPP\right]+\left[E-P\right]+\left[EI_{A}\right]+\left[EI_{A}I_{B}\right]$$

Taking into account that:

(A3) $$Ks=\frac{\left[E\right]\cdot \left[pNPP\right]}{\left[E•pNPP\right]}$$

(A4) $$K_{iA\;}=\frac{\left[E\right]\cdot \left[I_{A}\right]}{\left[EI_{A}\right]}$$

(A5) $$K_{iB}=\frac{\left[EI_{A}\right]\left[I_{B}\right]}{\left[EI_{A}I_{B}\right]}$$

Substituting the equations (4) and (5) into (2), and assuming initial velocity condition ([pNPP]_0_>>[E]_0_, so [pNPP]_0_ = [pNPP], we obtained:

(A6) $$\left[E\right]=\frac{{\left[E\right]}_{T}-\left[E\cdot pNPP\right]\cdot \left\{1+\frac{k_{2}}{k_{3}}\right\}}{\left\{1+\frac{\left[I_{A}\right]}{K_{iA}}+\frac{\left[I_{A}\right]\cdot \left[I_{B}\right]}{K_{iA}\cdot K_{iB}}\right\}}$$

Then, substituting (5) into (3), we obtained:

(A7) $$\left[E\cdot pNPP\right]=\frac{\frac{k_{3}\cdot {\left[E\right]}_{T}\cdot \left[pNPP\right]}{k_{2}+k_{3}}}{\frac{K_{s}\cdot k_{3}}{k_{2}+k_{3}}\cdot \left\{1+\frac{\left[I_{A}\right]}{K_{IA}}+\frac{\left[I_{A}\right]\left[I_{B}\right]}{\left[K_{IA}\cdot K_{IB}\right]}\right\}+\left[pNPP\right]}$$

Rate measured as production of [E-P]

$$v=\frac{\frac{k_{2}\cdot k_{3}}{k_{2}+k_{3}}\cdot \left\{{\left[E\right]}_{T}\cdot \left[pNPP\right]\right\}}{\frac{K_{s}\cdot k_{3}}{k_{2}+k_{3}}\cdot \left\{1+\frac{\left[I_{A}\right]}{K_{IA}}+\frac{\left[I_{A}\right]\left[I_{B}\right]}{\left[K_{IA}\cdot K_{IB}\right]}\right\}+\left[pNPP\right]}$$

where

$$K_{M}=\left(\frac{K_{s}\cdot k_{3}}{k_{2}+k_{3}}\right)$$

So, we obtained:

$$K_{M,\mathrm{app}}=K_{M}\cdot \left(1+\frac{\left[I_{A}\right]}{K_{IA}}+\frac{\left[I_{A}\right]\left[I_{B}\right]}{\left[K_{IA}\cdot K_{IB}\right]}\right)$$

Taking into account that

$$\left[I_{A}\right]=\left[I_{B}\right]\;K_{M,\mathrm{app}}=K_{M}\cdot \left(1+\frac{\left[I\right]}{K_{IA}}+\frac{{\left[I\right]}^{2}}{\left[K_{IA}\cdot K_{IB}\right]}\right)$$
