# Supplementary material for: Evidence of Insulin-Sensitizing and Mimetic Activity of the Sesquiterpene Quinone Avarone, a Protein Tyrosine Phosphatase 1B and Aldose Reductase Dual Targeting Agent from the Marine Sponge Dysidea avara
Source: Pharmaceutics. 2023 Feb 4;15(2):528. doi: 10.3390/pharmaceutics15020528 (PMC9964544; doi:10.3390/pharmaceutics15020528)
Supplement: Supplementary file 1 [file pharmaceutics-15-00528-s001.zip › pharmaceutics-2132861-supplementary.pdf]

Evidence of insulin-sensitizing and mimetic activity of the sesquiterpene quinone avarone, a Protein Tyrosine Phosphatase 1B and Aldose Reductase dual targeting agent from the marine sponge *Dysidea avara*

Marcello Casertano [1†], Massimo Genovese [2†], Alice Santi [2], Erica Pranzini [2], Francesco Balestri [3,4], Lucia Piazza[3], Antonella Del Corso [3,4], Sibel Avunduk [5], Concetta Imperatore [1], Marialuisa Menna [1*], and Paolo Paoli [2*]

SUPPLEMENTARY MATERIAL

Figure S1. HRESI-MS spectrum of avarol (**1**) 1

Figure S2. ^{1}H NMR spectrum of avarol (**1**) in $CDCl_3$ (600 MHz) 1

Figure S3. HRESI-MS spectrum of avarone (**2**) 2

Figure S4. ^{1}H NMR spectrum of avarone (**2**) in $CDCl_3$ (600 MHz) 2

Figure S5. HRESI-MS spectrum of 3-methylaminoavarone (**3**) 3

Figure S6. ^{1}H NMR spectrum of 3-methylaminoavarone (**3**) in $CDCl_3$ (600 MHz) 3

Figure S7. HRESI-MS spectrum of 4-methylaminoavarone (**4**) 4

Figure S8. ^{1}H NMR spectrum of 4-methylaminoavarone (**4**) in $CDCl_3$ (600 MHz) 4

Figure S9. Determination of the IC_{50} values for PTP1B and for AKR1B1 5

Figure S10. Dilution assay 6

Figure S11. Dependence of K_M and V_{max} from avarone (**2**) concentration 6

Figure S12. Docking of compounds **1-5** and of *p*-benzoquinone 7

Figure S13. Binding sites of avarone (**2**) identified by docking analyses 8

Figure S14. Determination of the IC_{50} values of avarone for PTP1B (A), and TC-PTP (B) 8

Figure S15. Binding sites of avarone (**2**) identified by docking analyses with TC-PTP 9

Figure S16. Full western blot of avarone on insulin signaling pathway 10

Table S1. Binding free energies of compounds **1-5** and of *p*-benzoquinone to PTP1B and TC-PTP 11

Figure S1. HRESI-MS spectrum of avarol (**1**)

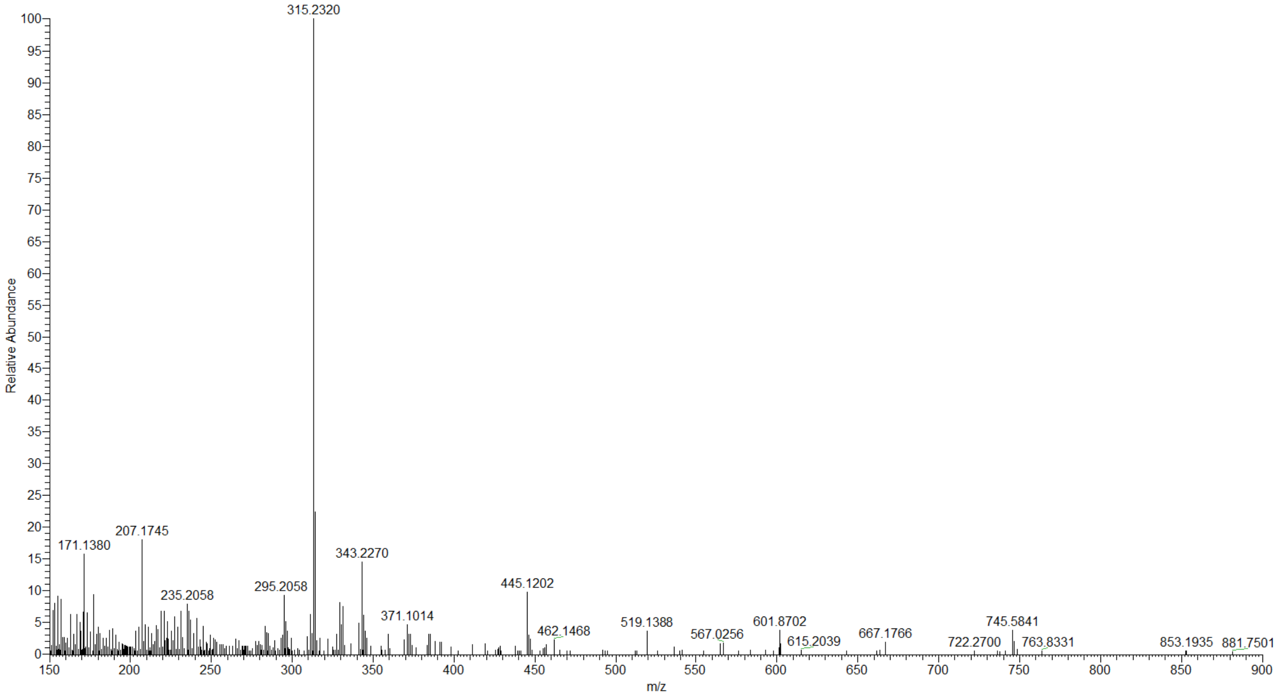

Figure S2. ^{1}H NMR spectrum of avarol (**1**) in $CDCl_3$ (600 MHz)

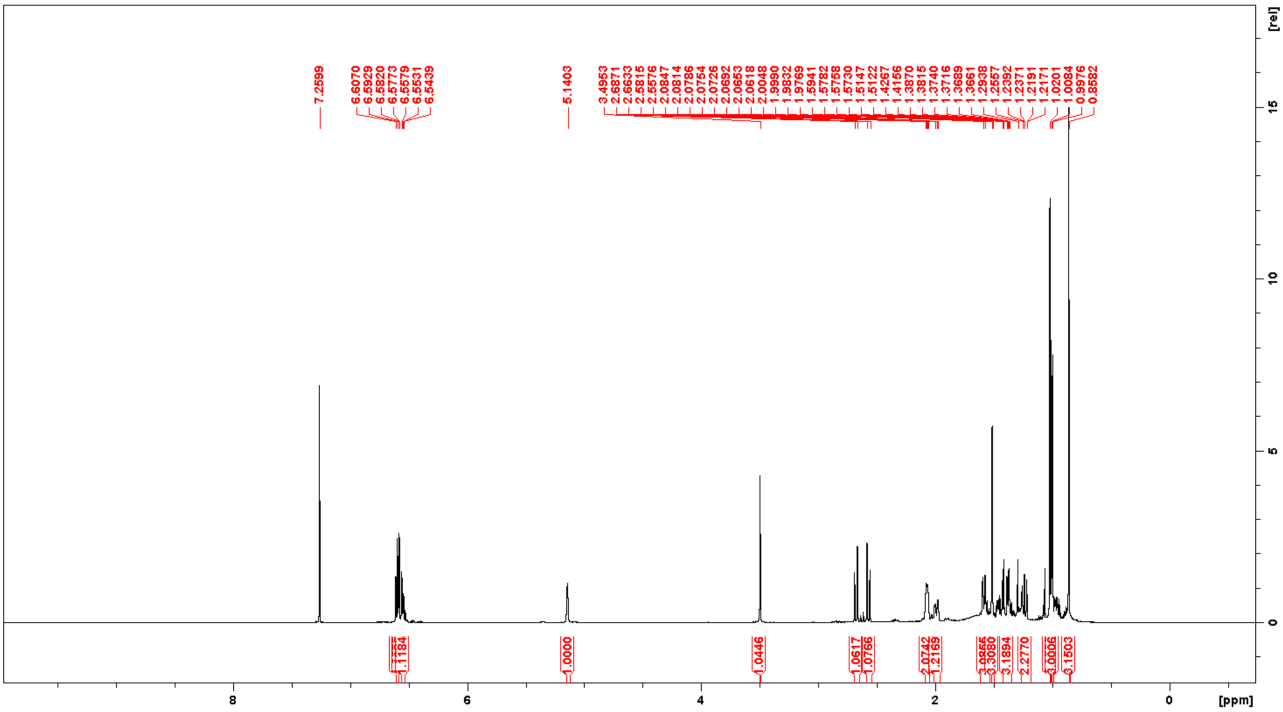

Figure S3. HRESI-MS spectrum of avarone (**2**)

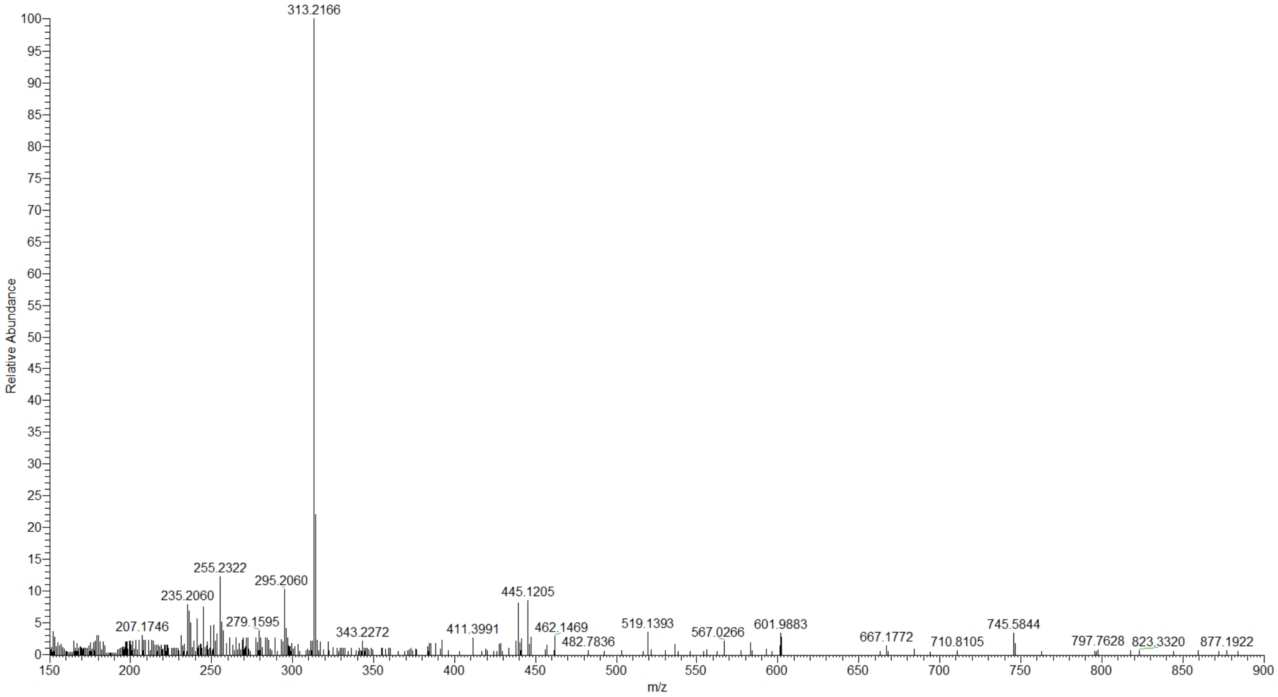

Figure S4. ^{1}H NMR spectrum of avarone (**2**) in $CDCl_3$ (600 MHz)

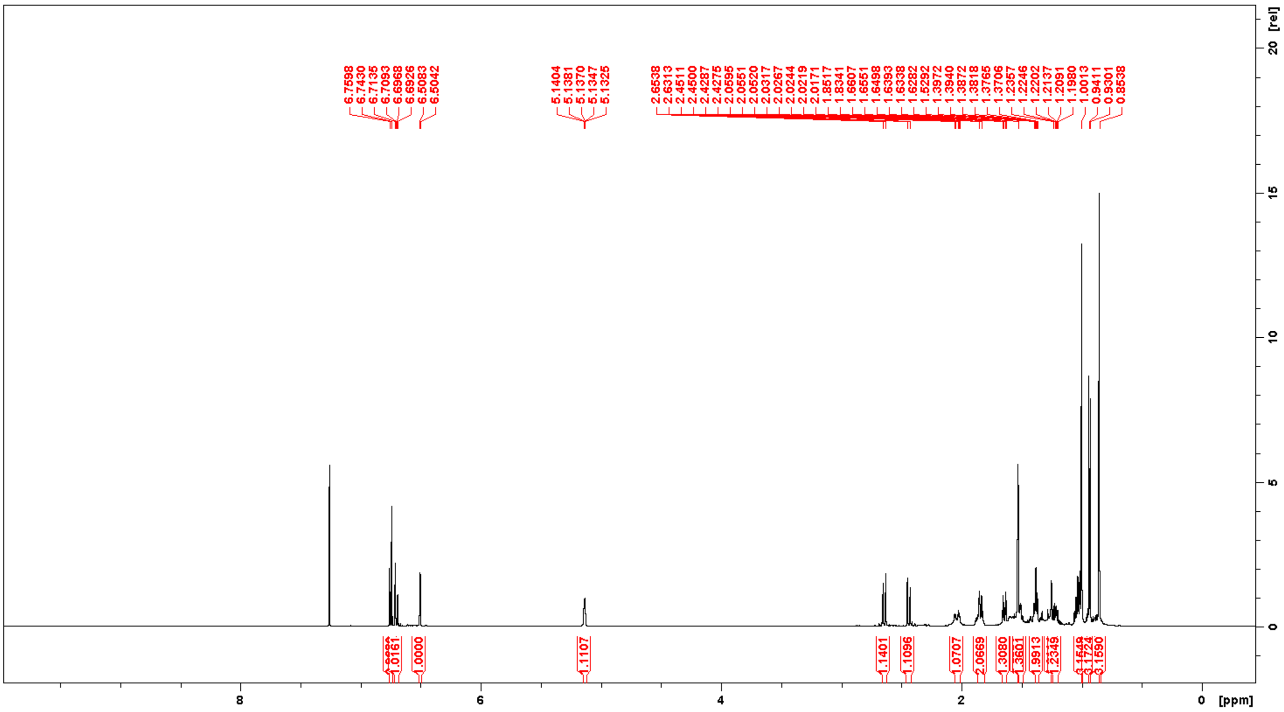

Figure S5. HRESI-MS spectrum of 3-methylaminoavarone (**3**)

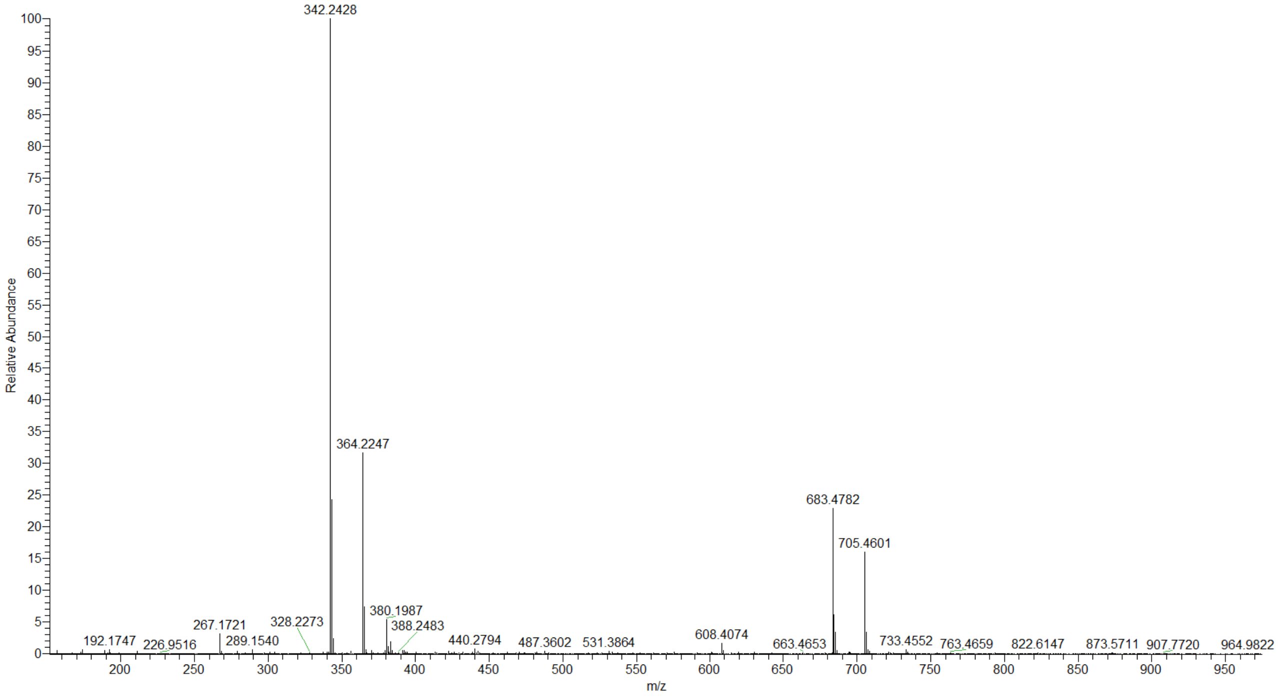

Figure S6. ^{1}H NMR spectrum of 3-methylaminoavarone (**3**) in $CDCl_3$ (600 MHz)

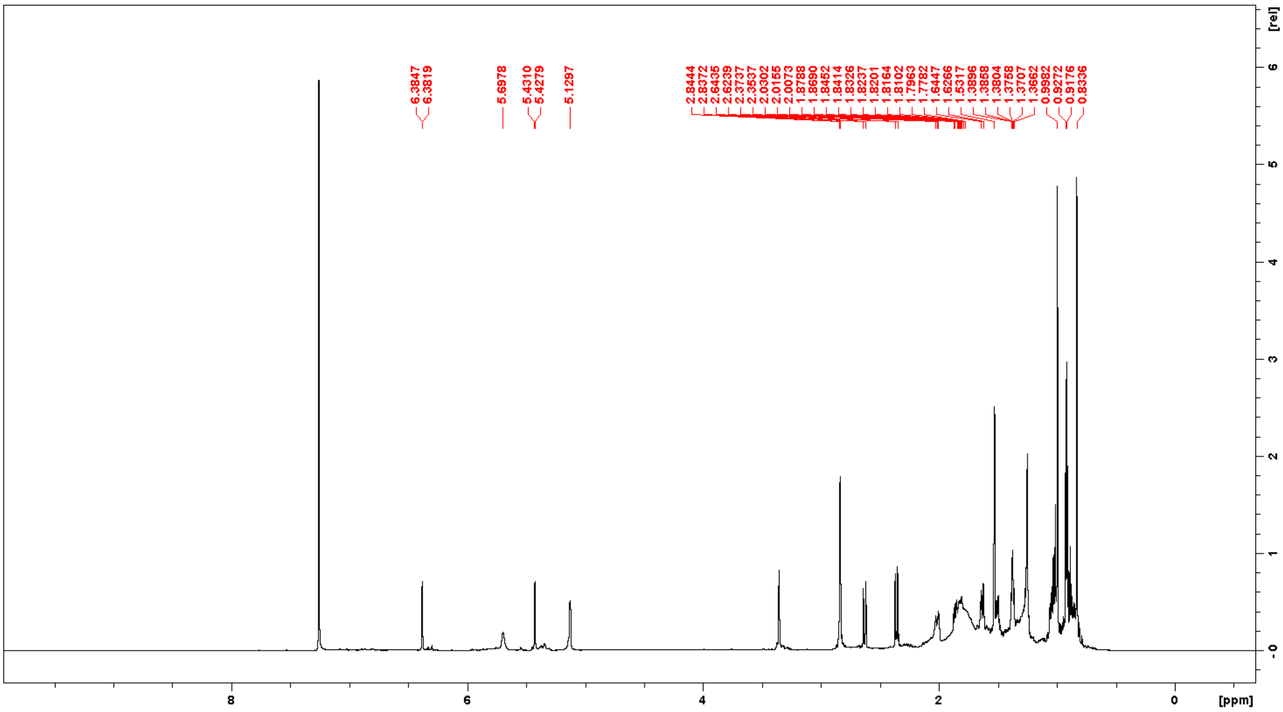

Figure S7. HRESI-MS spectrum of 4-methylaminoavarone (**4**)

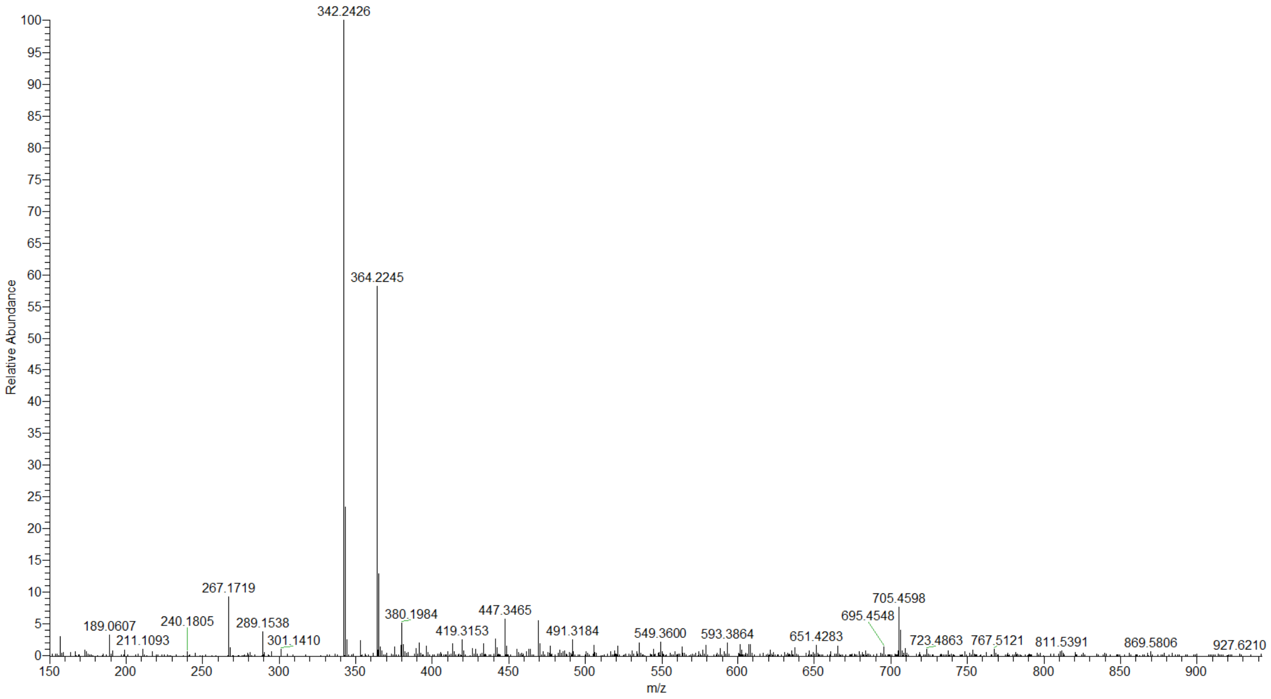

Figure S8. ^{1}H NMR spectrum of 4-methylaminoavarone (**4**) in $CDCl_3$ (600 MHz)

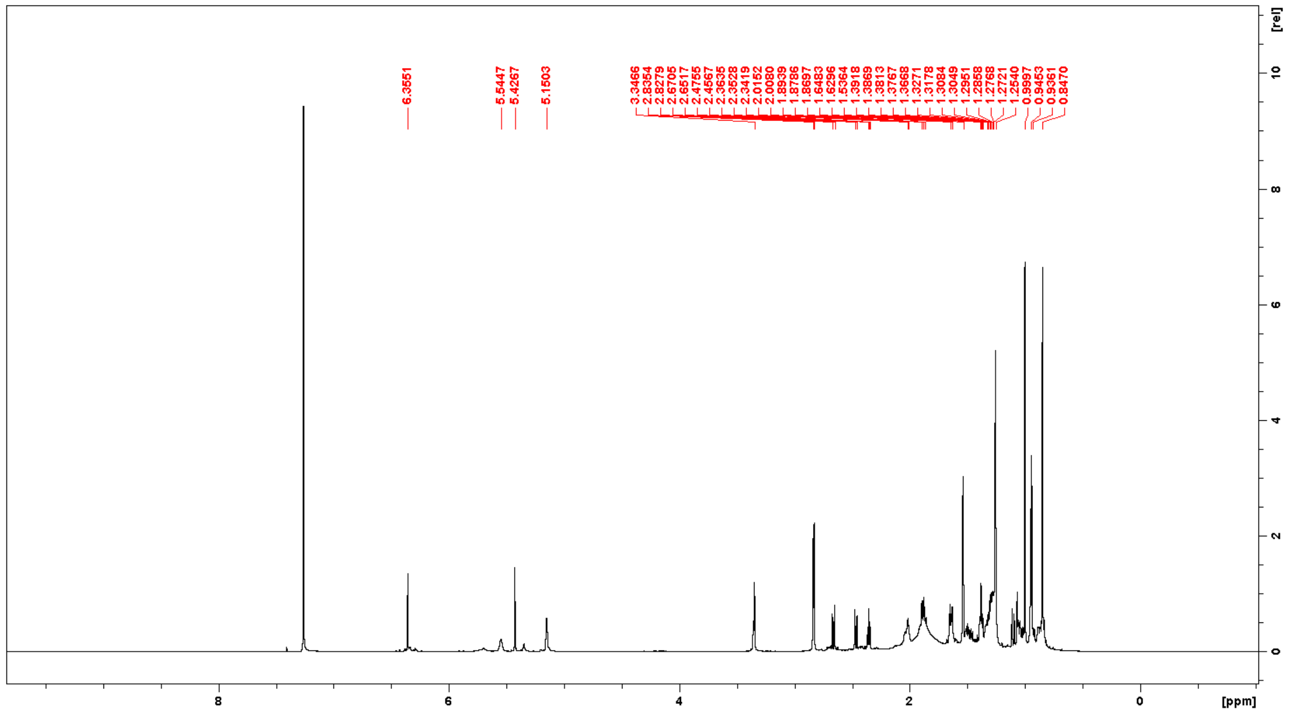

Figure S9. Determination of the IC_{50} values for A) PTP1B and for B) AKR1B1

A)

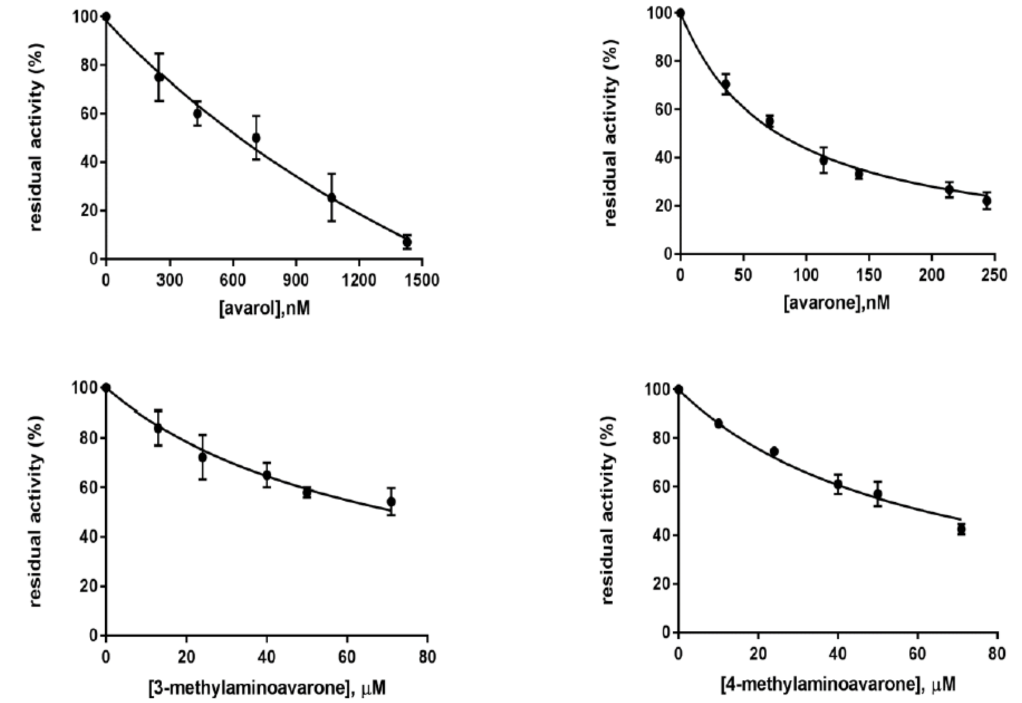

B)

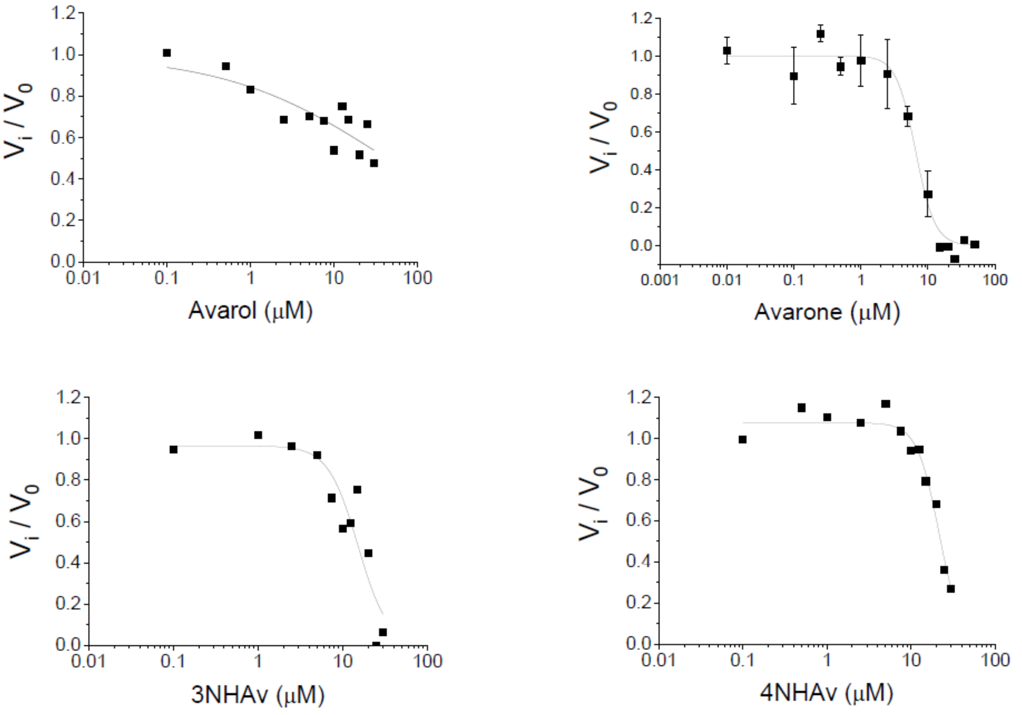

Figure S10. Dilution assay. An aliquot of PTP1B was incubated for 1 h at 37°C in the presence of saturating concentration (50 μM) of avarone (**2**). A control test was prepared diluting PTP1B solution with the same volume of DMSO, the solvent used to dissolve **2**. After 1 h, an aliquot of samples was withdrawn and diluted 400 fold in buffer assay to evaluate the residual activity of the enzyme. All tests were carried out in triplicate. Data obtained were normalized respect to control sample. Data showed in the figure suggested that avarone behaved as a reversible inhibitor of PTP1B.

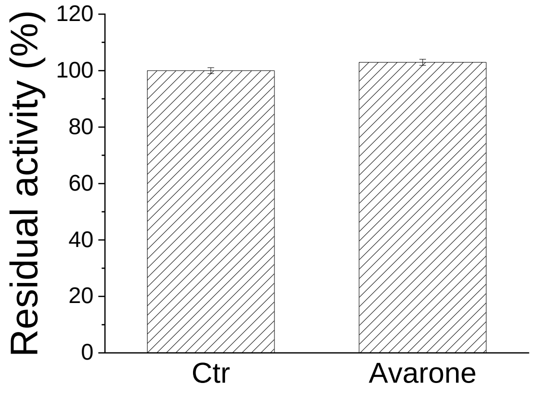

Figure S11. Dependence of K_M and Vmax from avarone (**2**) concentration. The concentration of avarone used were: 0, 4.0, 5.5, and 7.0 μM. To calculate both K_M and V_{max} valued, we determined the initial hydrolysis rate at seven different substrate (pNPP) concentrations (0.5, 1, 2 4, 8 16, 25 mM). Experimental data were fitted with Michaelis-Menten equation using OriginPro 2021 software

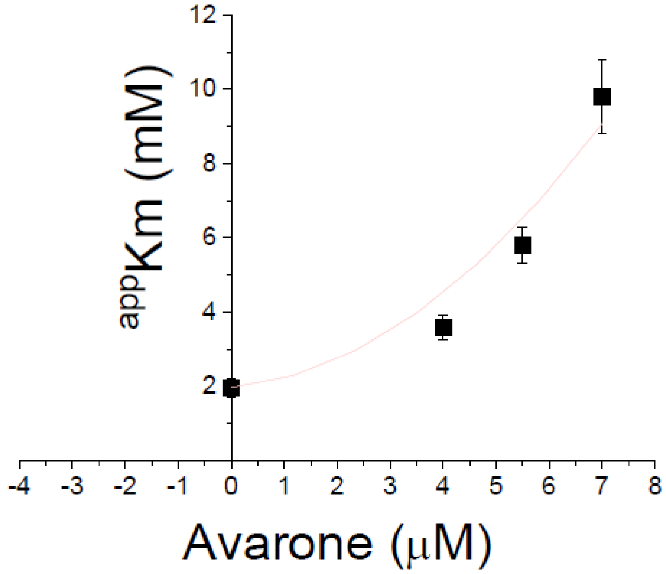

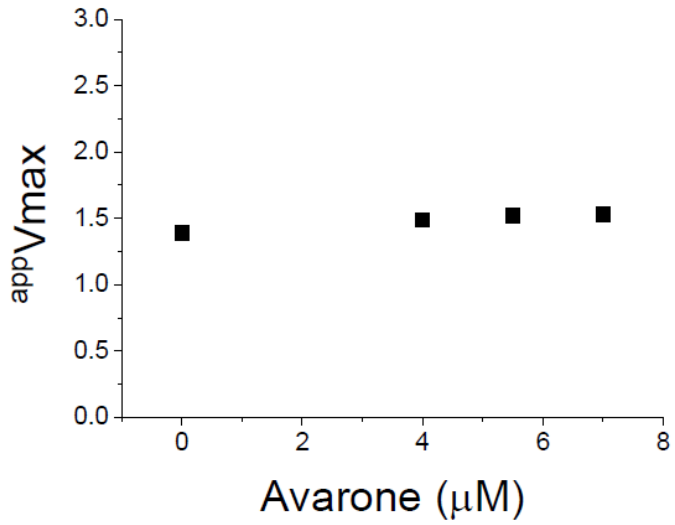

Figure S12. Docking analyses of the avarol (**1**), avarone (**2**), 3-methylaminoavarone (**3**), 4-methylaminoavarone (**4**), p-benzoquinone and dysidine (**5**) into the active site of PTP1B. Images of compounds docked into active site of PTP1B were obtained using UCSF Chimera. Moreover, 2D representations of interactions were performed using Ligplot plus

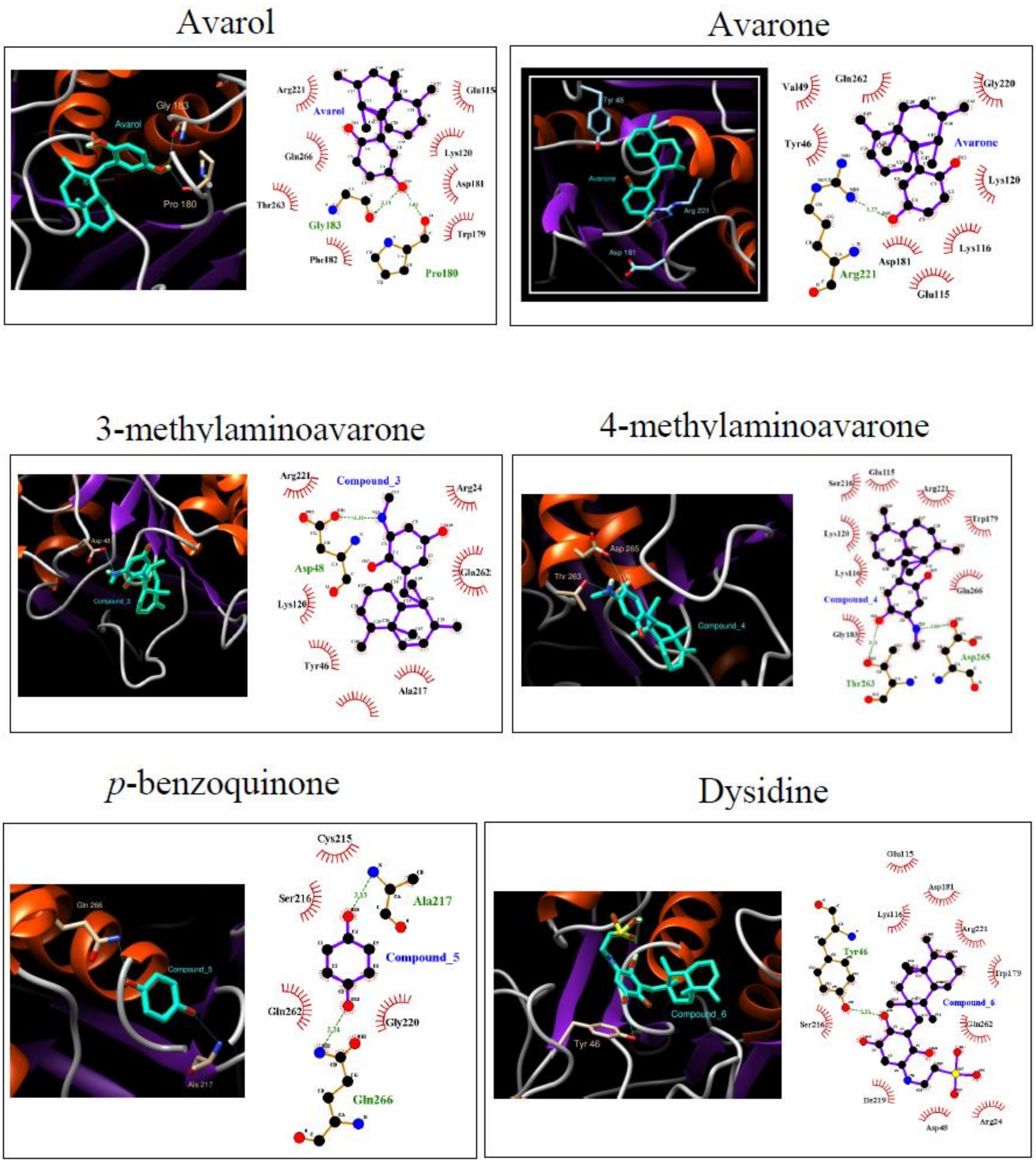

Figure S13. Binding sites of avarone (**2**) identified by docking analyses. (A), General view of interaction sites of avarone on PTP1B surface. (B), detail showing the molecules of avarone bound into the active site of PTP1B. The images were obtained using the UCSF Chimera software.

A **B**

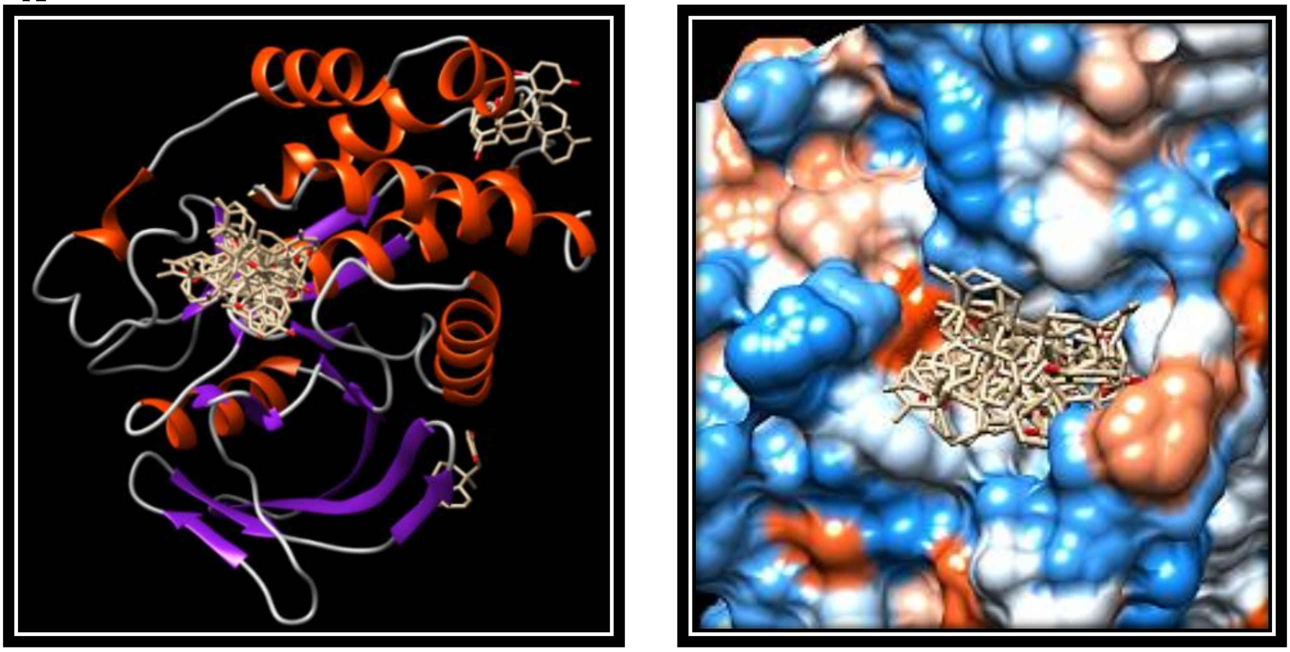

Figure S14. Determination of the IC_{50} values of avarone for PTP1B (A), and TC-PTP (B).

A **B**

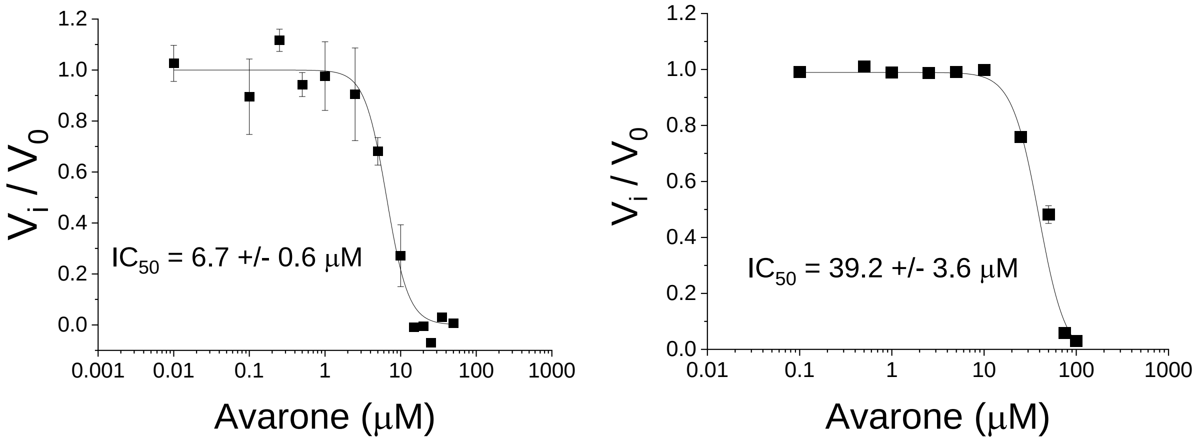

Figure S15. Binding sites of avarone identified by docking analyses with TC-PTP. (A), General view of interaction sites of avarone on TC-PTP surface. To perform the docking analysis, the 7F5N structure deposited in the PDB database was used (B, C and D), detailed images showing the first three energetically favorable poses of avarone bound into the active site of TC-PTP. Images of avarone docked into active site of TC-PTP were obtained using UCSF Chimera. Moreover, 2D representations of interactions were performed using Ligplot plus.

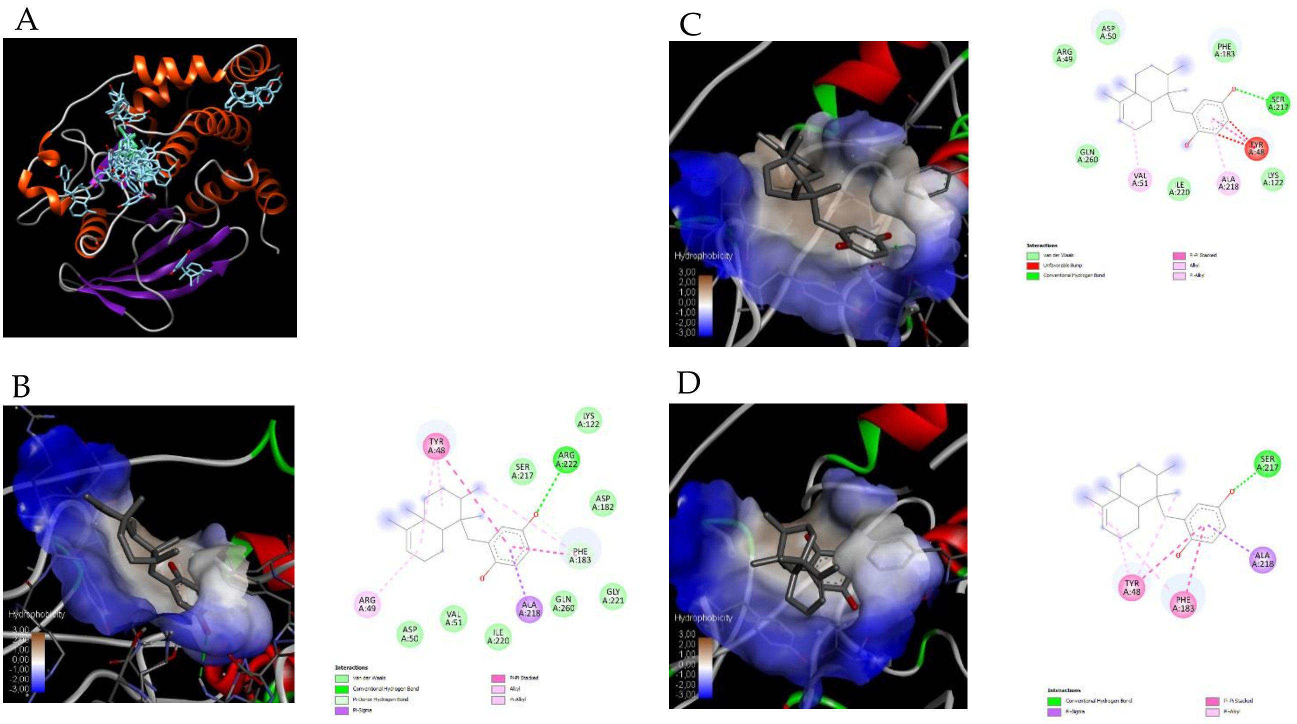

Figure S16. Full western blot of avarone on insulin signaling pathway. C2C12 cells were serum-starved and stimulated with 10 nM insulin (Ins), 25 µM of avarone (Ava) or with a combination of both (Ins+Ava) for 30 min at 37°C.

p-Akt (1)

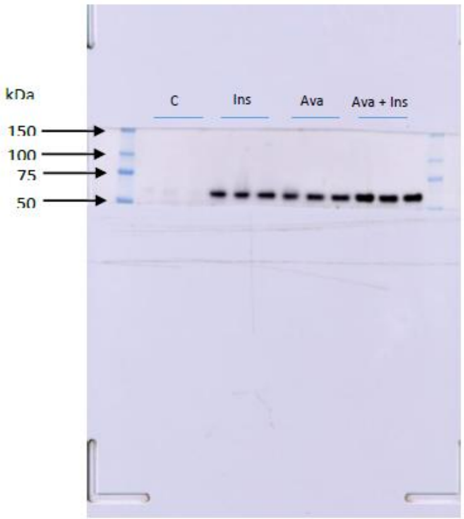

Actin (1)

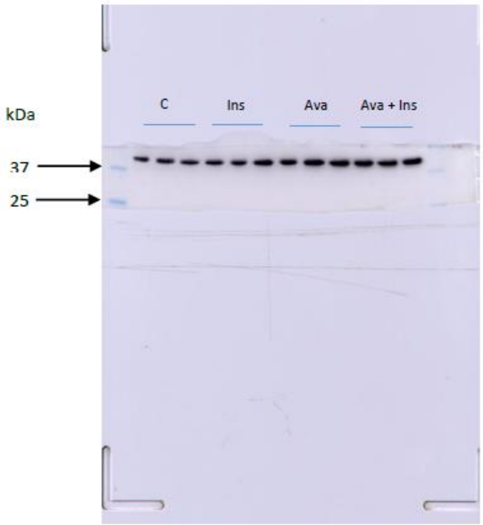

Akt tot (2)

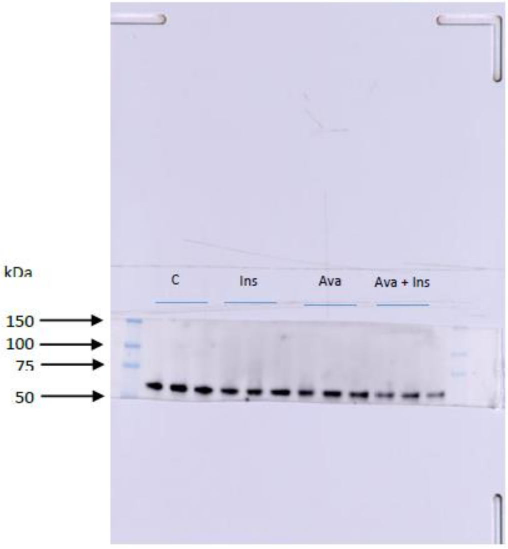

Actin (2)

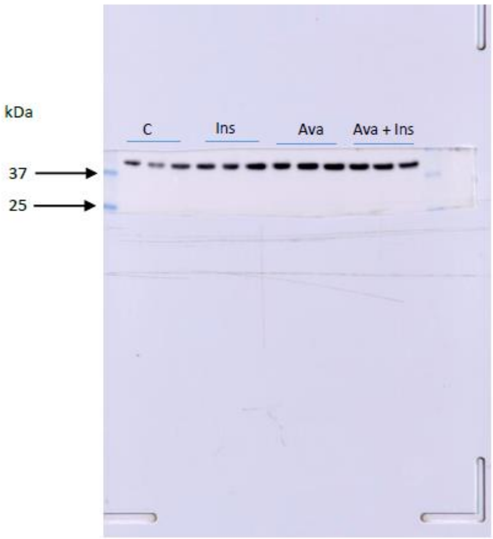

Table S1. Binding free energies of compounds **1-5** and of p-benzoquinone to PTP1B and TC-PTP. Table show the ΔG° values for both enzymes calculated using AutoDock Vina software

**Protein**	**PTP1B (△G°) kcal/mol**	**TC-PTP (△G°) kcal/mol**
avarol	-7.4	------
avarone	-7.8	-7.2
3-methylaminoavarone	-6.4	------
4-methylaminoavarone	-6.7	------
p-benzoquinone	-3.6	------
dysidine	-6.7	------
